# Polymorphism-Dependent Dynamic Ultralong Organic Phosphorescence

**DOI:** 10.34133/2020/8183450

**Published:** 2020-02-07

**Authors:** Mingxing Gu, Huifang Shi, Kun Ling, Anqi Lv, Kaiwei Huang, Manjeet Singh, He Wang, Long Gu, Wei Yao, Zhongfu An, Huili Ma, Wei Huang

**Affiliations:** ^1^Institute of Flexible Electronics (IFE), Northwestern Polytechnical University (NPU), 127 West Youyi Road, Xi'an 710072, China; ^2^Key Laboratory of Flexible Electronics (KLOFE) & Institute of Advanced Materials (IAM), Nanjing Tech University (NanjingTech), 30 South Puzhu Road, Nanjing 211816, China

## Abstract

Developing ultralong organic phosphorescence (UOP) materials with smart response to external stimuli is of great interest in photonics applications, whereas the manipulation of molecular stacking on tuning such dynamic UOP is still a formidable challenge. Herein, we have reported two polymorphs with distinct photoactivated dynamic UOP behavior based on a pyridine derivative for the first time. Our experiment revealed that the dynamic UOP behavior including photoactivation and deactivation feature is highly dependent on irradiation intensity and environmental atmosphere. Additionally, given the unique dynamic UOP feature, these phosphors have been successfully applied to phosphorescence-dependent molecular logic gate and timing data storage. This result not only paves a way to design smart functional materials but also expands the scope of the applications on organic phosphorescence materials.

## 1. Introduction

Stimuli-responsive materials, that is, smart materials, whose physical properties can be controllably tuned by external stimuli, such as heat, light, pressure, and solvent, are of typical interest because of their promising potential applications [1, 2]. For instance, equipped with the sensibility of one or more external stimuli, the photoluminescent materials can be applied to diverse fields ranging from biological detection [3, 4] and sensors [5, 6] to optical memory devices [7–9] and logic gates [10, 11]. Despite great potentials in practical applications, it remains a formidable challenge to develop such smart materials so far. Recently, a new type of organic luminescent materials with ultralong organic phosphorescence (UOP) has drawn considerable attentions owing to their distinctive advantages of long-lived persistent luminescence and high exciton utilization [12–15]. A library of UOP luminogens has been developed with a series of feasible strategies including crystal engineering [16–23], H-aggregation [24–28], host-guest doping [29–32], and so on [33–44], which mainly show steady-state phosphorescence emission at room temperature. Very recently, a dynamic photoactivated UOP was fortuitously found in triazines and phenothiazine derivatives under ambient conditions [45–48]. Namely, the phosphorescence lifetime can be rationally regulated by photoactivation time. Notably, crystallization is essential to such phenomenon, implying the importance of molecular stacking on dynamic UOP. Moreover, molecular stacking has been proved to be effective in manipulating luminescent properties of organic materials in solid state [49–52]. Therefore, it is urgent to provide an insight from molecular stacking to understanding the inherent mechanism in dynamic UOP.

## 2. Results

### 2.1. Culture and Observation of Polymorphs

**Figure 1:**
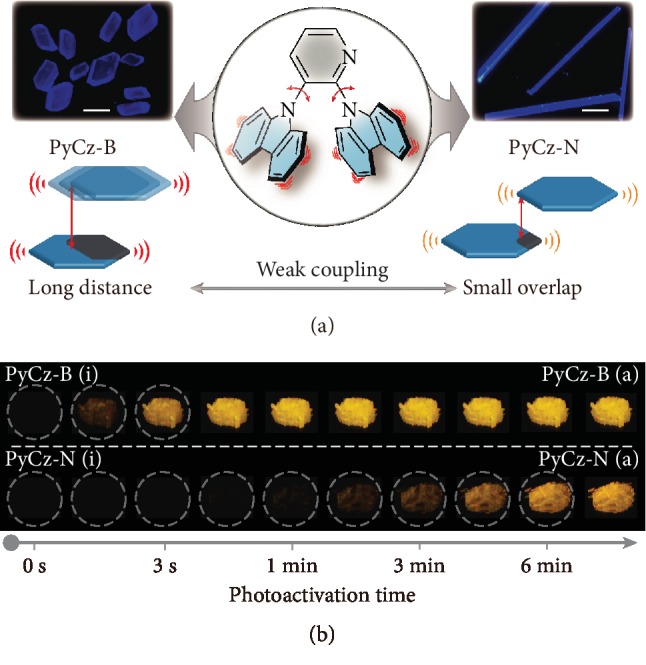
The structure of PyCz and two kinds of crystal of PyCz with different photoactivation UOP speed. (a) Molecular structure of PyCz with two kinds of molecular packing mode. Insets: the fluorescence microscopy images of PyCz-B and PyCz-N with the scale bar of 200 *μ*m. (b) The afterglow photos of PyCz-B and PyCz-N at different photoactivation time with a handheld 365 nm UV lamp (40 mW/cm^2^) under ambient conditions, and the dashed circles are used to distinguish the samples with low afterglow intensity.

Two polymorphs of PyCz, PyCz-B (block-type crystal) and PyCz-N (needle-like crystal), were cultured by slow solvent evaporation from different binary solvents under ambient condition. The binary solvents are ethyl acetate and *n*-hexane (V_EA_ : V_Hex_ = 3 : 2) for PyCz-B and dichloromethane and *n*-hexane (V_DCM_ : V_Hex_ = 3 : 2) for PyCz-N, respectively. The fluorescence microscopy images of these two crystalline polymorphs are depicted in Figure 1(a). Both polymorphs showed blue emission upon UV irradiation and after switching off the UV lamp under ambient conditions; no visible persistent luminescence have been observed. Impressively, both PyCz-B and PyCz-N demonstrated distinct persistent luminescence after long photoactivation for a period of time under UV light irradiation. Observationally, PyCz-B showed bright persistent luminescence that lasted for several seconds, activated by strong 365 nm light (40 mW/cm^2^) within 3 s, while it took much longer for the photoactivation of PyCz-N, about 6 min (Figure 1(b) and [Supplementary-material supplementary-material-1]).

### 2.2. Photophysical Properties

**Figure 2:**
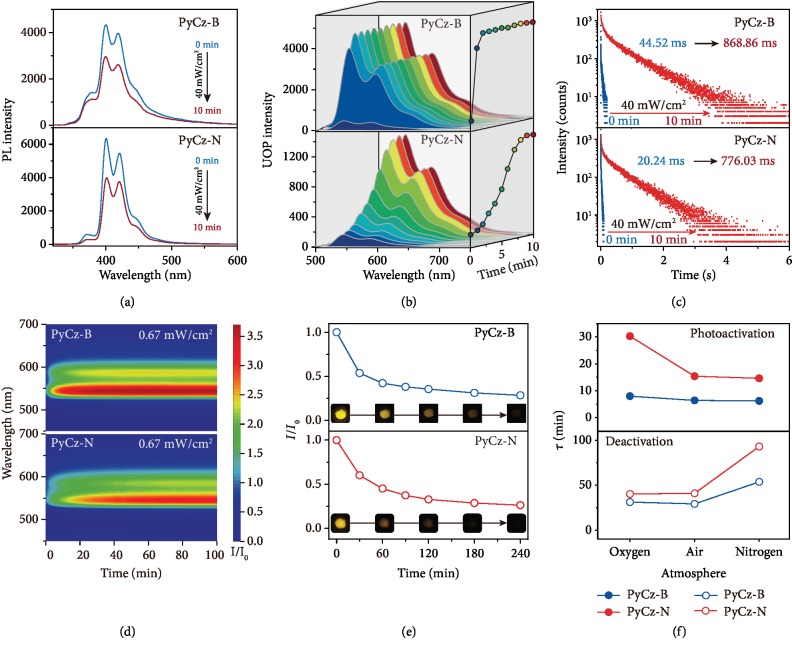
Photophysical properties of PyCz under ambient conditions. (a) Steady-state PL spectra of PyCz-B and PyCz-N before (blue line) and after (red line) long photoactivation (power = 40 mW/cm^2^, time = 10 min) under ambient conditions. (b) The phosphorescence spectra and the corresponding peak intensity at 544 nm of PyCz-B and PyCz-N after different photoactivation time ranging from 0 to 10 min by a 365 nm lamp (power = 40 mW/cm^2^) under ambient conditions. (c) Lifetime decay profiles of the emission peak at 544 nm of PyCz-N and PyCz-B before and after long photoactivation under ambient conditions. (d) The time-phosphorescence mapping of PyCz-B and PyCz-N for 100 min with the 365 nm light source power of 0.67 mW/cm^2^ under ambient conditions. (e) The phosphorescence variation value (*I*/*I*_0_) at 544 nm during the deactivation of the UOP for PyCz-N and PyCz-B after long photoactivation under ambient conditions. Insets: the corresponding photos at different deactivation time. (f) The corresponding dynamic lifetime *τ* for the photoactivation by weak 365 nm light (power = 0.67 mW/cm^2^) and deactivation of PyCz-B and PyCz-N under different atmosphere.

To understand the different dynamic optical properties of both polymorphs, the photophysical properties of PyCz phosphors in the crystal were investigated under ambient conditions. As shown in Figure 2(b), PyCz-B and PyCz-N both have weak phosphorescence signals in their initial states. After long photoactivation (365 nm light with the power of 40 mW/cm^2^ for 10 min), the phosphorescence intensity was largely enhanced by more than 6 times with little changes in emission peaks at 544 and 588 nm, accompanied by a slight decrease in fluorescence intensity and lifetime (Figures 2(a) and [Supplementary-material supplementary-material-1] and [Supplementary-material supplementary-material-1]). Simultaneously, the lifetimes of the emission bands at around 544 nm were prolonged from 44.52 to 868.86 ms and 20.24 to 776.03 ms for PyCz-B and PyCz-N, respectively (Figures 2(c) and [Supplementary-material supplementary-material-1] and [Supplementary-material supplementary-material-1]), suggesting photoactivated dynamic UOP behavior for both polymorphs. These dynamic UOP features were further confirmed by the prolonged emission lifetime (from 365.53 to 965.96 ms in PyCz-B and 208.50 to 1065.46 ms in PyCz-N) in nitrogen during the long photoactivation ([Supplementary-material supplementary-material-1] and [Supplementary-material supplementary-material-1]). Such dynamic UOP behavior also occurred under the irradiation of weak UV light. As shown in Figure 2(d), the phosphorescence intensity enhanced slowly for both PyCz-B and PyCz-N. The photoactivation took place within 20 min for PyCz-B and >60 min for PyCz-N after irradiation by weak 365 nm light with excitation intensity of 0.67 mW/cm^2^. The enhancement ratio (*I*/*I*_0_) of phosphorescence intensity was 3.7 and 3.1 for PyCz-B and PyCz-N, respectively. In addition, this dynamic UOP behavior with different photoactivation speed was also observed by using excitation intensities of 0.56 and 0.39 mW/cm^2^ ([Supplementary-material supplementary-material-1] and [Supplementary-material supplementary-material-1]). Notably, such a dynamic behavior of PyCz was reversible. The activated states of both polymorphs (PyCz-B(a) and PyCz-N(a)) can gradually return to their initial states (PyCz-B(i) and PyCz-N(i)) within a period of time when they were kept in the dark (Figure 2(e)), and such reversibility can be repeated for many times ([Supplementary-material supplementary-material-1]).

### 2.3. Experimental Investigation

To gain a deeper insight into the dynamic UOP behavior, the photophysical parameters of both polymorphs before and after photoactivation were calculated and tabulated in [Supplementary-material supplementary-material-1]. It was found that the nonradiative decay rates in the activated state (1.15 and 1.29 s^−1^) are far less than those in the initial state (21.74 and 50.00 s^−1^) for both polymorphs (PyCz-B and PyCz-N, respectively), which are responsible for the prolonged UOP lifetime. Although, this decay rate is well-known to be related to quenching factor and molecular motions. Therefore, this is the first time we have studied the dynamic processes under different atmospheres to reveal the effect of the quenching factor from the external environment for two polymorphs (Figures 2(f), [Supplementary-material supplementary-material-1], and [Supplementary-material supplementary-material-1]). With the alteration of atmosphere from oxygen to nitrogen, the lifetime (*τ*) for dynamic UOP behavior of both polymorphs have followed the same trend: the *τ* of photoactivation was shortened while it was prolonged for the deactivation process. Notably, PyCz-N shows wider *τ* rangeability, indicating that PyCz-N is more sensitive to the atmosphere. Besides, it is easily found that no matter how the atmosphere changed, the *τ* of PyCz-B was always smaller than that of PyCz-N under the same condition. So, we concluded that the atmosphere is not the cause for the different dynamic UOP feature of PyCz-B and PyCz-N.

**Figure 3:**
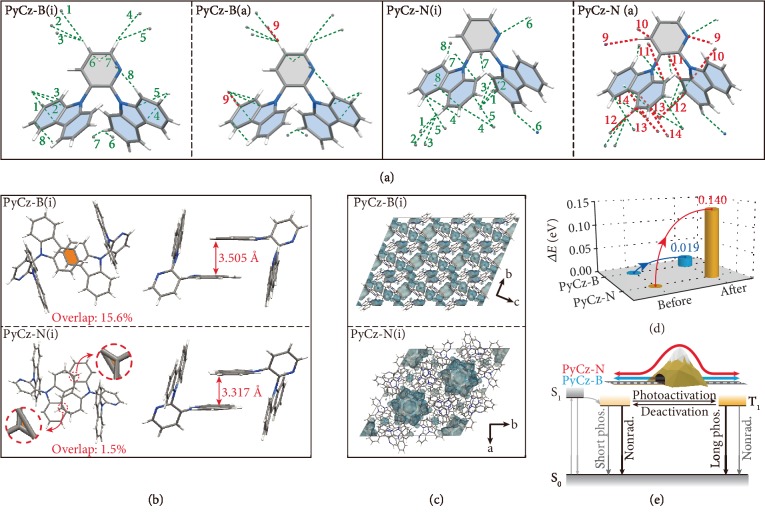
Crystal stacking analysis and simulated calculations for dynamic ultralong organic phosphorescence of PyCz. (a) The intermolecular interactions around one molecule in PyCz-B and PyCz-N before and after long photoactivation in crystalline state measured at 100 K, the green dash line refers to the initial interactions and the red dash line is the added interactions after long photoactivation. (b) The *π*-*π* overlap and distance of selected dimer with *π*-*π* interactions in PyCz-B(i) and PyCz-N(i). Note that the green isosurface refer to the calculated molecular interactions by IGM, the isovalue is 0.008. (c) The free volume region (cyan isosurface) in single crystal cells of PyCz-B(i) and PyCz-N(i). (d) The calculated change of single molecular energy in PyCz-B and PyCz-N during the process of photoactivation. (e) Proposed mechanism for different dynamic speeds of dynamic UOP in polymorphs.

When PyCz crystals were kept in liquid nitrogen environment (77 K), the dynamic UOP behavior was almost insensitive to the photoactivation time, while the deactivation process was prolonged. Especially, the deactivation time in PyCz-N at 77 K (>72 h) is longer than that at 298 K (~4 h) ([Supplementary-material supplementary-material-1]). These phenomenon could be ascribed to the suppression of molecular motions. Furthermore, the single-crystal analysis of both polymorphs was conducted before and after long photoactivation. In the initial state, the molecule in PyCz-N(i) was confined by multiple molecular interactions, including *π*···*π* (3.378 Å, 3.400 Å), C-H···*π* (2.701-2.873 Å), and C-H···N (2.633 Å) interactions, while there is only C-H···*π* (2.732-2.849 Å) interactions in PyCz-B(i). After long photoactivation, obviously, molecular motions happened in the crystal and the distances of intermolecular interactions in PyCz-N(a) decreased by 0.019-0.025 Å, accompanied by newly formed *π*···*π* (3.388-3.399 Å) and C-H···N (2.748 Å) interactions. The decrease in intermolecular distances for PyCz-B(a) is smaller (0.002-0.009 Å) as compared to PyCz-N(a), along with a new C-H···*π* (2.895 Å) interaction formation (Figure 3(a) and Tables [Supplementary-material supplementary-material-1] and [Supplementary-material supplementary-material-1]). With the enhancements of intermolecular interactions in both polymorphs, nonradiative transition could be suppressed, thus leading to longer emission lifetimes. Additionally, through the analysis of the independent gradient model (IGM) [53, 54], the molecular interactions could be directly displayed by the isosurface in [Supplementary-material supplementary-material-1]. After long photoactivation, the *π*-*π* couplings and molecular interactions in both polymorphs have enhanced with larger isosurface, further proving that molecular motions have restricted for prolonging emission lifetimes after long photoactivation.

### 2.4. Crystal Stacking Analysis and Simulated Calculations

The molecular stacking may account for the different dynamic UOP behavior of PyCz polymorphs. As shown in Figure 3(b), both polymorphs before photoactivation have only weak *π*-*π* couplings, which are not favorable for phosphorescent emission [17, 42]. Specifically, the *π*-*π* distance in PyCz-B (3.505 Å) is larger than that in PyCz-N (3.317 Å), while the *π*-*π* overlap in PyCz-N (1.5%) is smaller than that in PyCz-B (15.6%). Moreover, the isosurface with the same isovalue (0.008) in PyCz-N is much larger than that in PyCz-B, indicating the stronger intermolecular interactions. These results indicated that the weaker *π*-*π* interactions caused by long *π*-*π* distance may account for faster dynamic UOP in PyCz-B. To gain more insight into the interrelation between dynamic UOP and molecular stacking, a set of theoretical calculations was carried out. Firstly, the free volume distributions in PyCz-B and PyCz-N were calculated, which provides the space for molecular motions to rationally manipulate the nonradiative transitions during photoactivation. From Figure 3(c), it was found that the unoccupied spaces distributed more dispersedly around the molecules in PyCz-B, while in PyCz-N, the unoccupied spaces mostly concentrated in the cavities with a little space left around the molecules. Thus, molecules in PyCz-B can adjust their configurations more easily and took less time to reach the activated states during photoactivation. Besides, the proportion of unoccupied spaces of PyCz-B and PyCz-N become smaller after long photoactivation, from 12.19% to 12.06% and 16.86% to 16.40%, respectively ([Supplementary-material supplementary-material-1] and [Supplementary-material supplementary-material-1]), further proving that the molecular stacking become tighter. In addition, the single molecular energy was calculated to characterize the energy variation from the initial state to the activated state. As illustrated in Figure 3(d), the energy increased by 0.019 and 0.140 eV for PyCz-B and PyCz-N after long photoactivation, respectively. In other words, there existed a larger energy barrier to overcome for PyCz-N molecules during the photoactivation. Owing to the smaller variation of single molecular energy, PyCz-B can be activated or deactivated much more easily than PyCz-N, verifying the difference in dynamic performance from the energy aspect (Figure 3(e)). Taken together, we speculated that the different contribution of nonradiative transitions by manipulating different molecular stacking lead to distinct dynamic UOP features in PyCz polymorphs.

### 2.5. Applications

**Figure 4:**
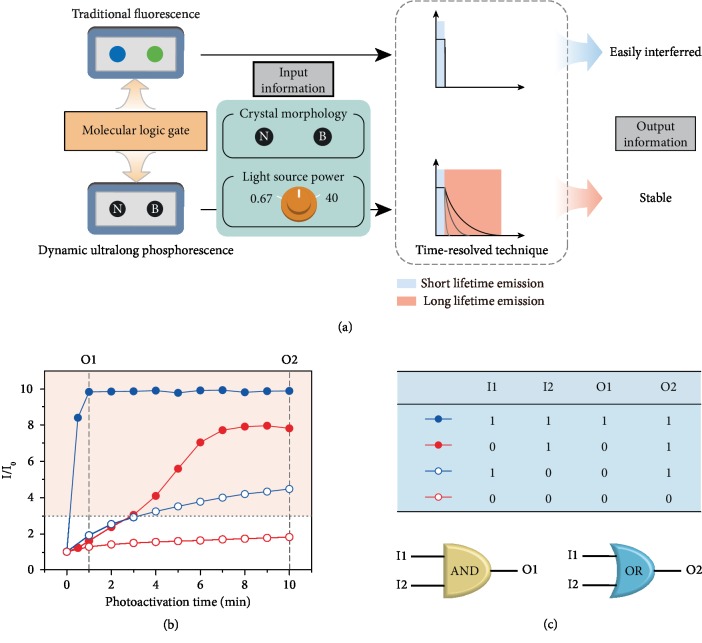
The demonstration of the application in molecular logic gate. (a) The model of molecular logic gate and the contrast between fluorescence logic gate and phosphorescence logic gate. (b) Under ambient conditions, the photoactivation of PyCz by different power UV light: PyCz-B activated by UV light with the power of 40 mW/cm^2^ and 0.67 mW/cm^2^ (blue line with solid and open circle, respectively), PyCz-N activated by UV light with the power of 40 mW/cm^2^ and 0.67 mW/cm^2^ (red line with solid and open circle, respectively). (c) The truth table for O1 and O2 and the proposed logic gate for PyCz with two inputs and two outputs.

Nowadays, integration of multiple molecular logic gates to construct molecular computers is particularly challenging mainly due to connectivity of molecular logic gates. In optical devices, it is much harder to establish complex logic gates because the light signals were easily disturbed. Regarding the superiority of the ultralong emission lifetime for elimination of background fluorescence interference by time-resolved technique [55–57], we here attempted to utilize this dynamic UOP material for molecular logic gate (Figure 4(a)). From Figure 4(b), it could be easily found that the dynamic UOP property of PyCz polymorphs is highly dependent on crystal morphology and power of irradiation light source. With fixed power of UV light irradiation, the photoactivation speed of PyCz-B was faster than that of PyCz-N under the same condition. Meanwhile, the stronger power of irradiation can greatly accelerate the photoactivation process. On the basis of abovementioned logic channels for rationally controlling the dynamic UOP, a molecular logic gate with two inputs and two outputs was fabricated. The crystal morphology (I1: PyCz-B is “1”, PyCz- N is “0”) and the light power (I2: 40 mW/cm^2^ is “1”, 0.67 mW/cm^2^ is “0”) were defined as inputs, the relative intensity of phosphorescence (*I*/*I*_0_) at 1 min and 10 min was selected as output 1 (O1) and output 2 (O2), respectively. If the phosphorescent intensity (*I*) is 3 times more than the initial intensity (I_0_), the output is “1.” Otherwise, the value is “0.” Thus, O1 and O2 can be recognized as the AND and OR phosphorescence-dependent molecular logic gate based on the truth table (Figure 4(c)). Additionally, with the reversibility of dynamic UOP (Figures [Supplementary-material supplementary-material-1] and [Supplementary-material supplementary-material-1]), PyCz-N can also be used for rewritable timing data storage ([Supplementary-material supplementary-material-1], [Supplementary-material supplementary-material-1] and [Supplementary-material supplementary-material-1]). The information can be stored for over 6 hours and can be rewritten by long photoactivation again. To the best of our knowledge, this is the first UOP example for molecular logic gate and rewritable timing data storage.

## 3. Discussion

In summary, we have developed two crystalline polymorphs of PyCz molecule, which showed distinct dynamic UOP behaviors owing to the different molecular stacking in crystal. PyCz-B showed much faster dynamic process than PyCz-N under the same conditions. Besides, it was found that the dynamic UOP behavior including photoactivation and deactivation features were highly dependent on irradiation intensity and environmental atmosphere for PyCz-B and PyCz-N. On the basis of experimental results and theoretical calculations, we speculated that the regulation of nonradiative transition through the manipulation of intermolecular stacking played a critical role in realizing various dynamic UOP behaviors. Given the fascinating optical features responding to multiple conditions, PyCz was successfully applied in phosphorescence-dependent molecular logic gate and timing data storage. This finding not only gives deeper understanding in photoactivatable dynamic UOP materials but also expands the scope of the applications of UOP materials.

## 4. Materials and Methods

### 4.1. Crystal Cultivation

Two types of crystals were prepared through slow solvent evaporation from different solutions. For PyCz-B, 50 mg PyCz was refluxed and dissolved in 3 mL ethyl acetate; then, 2 mL *n*-hexane was slowly injected over the solution; then, the solution was kept under ambient conditions; the block-like transparent crystal was incubated after four days. For PyCz-N, 50 mg PyCz was dissolved in 3 mL CH_2_Cl_2_; then, 2 mL *n*-hexane was slowly injected over the solution; then, the solution was kept under ambient conditions; the needle-like transparent crystal was obtained after two days.

### 4.2. Measurements

Nuclear magnetic resonance (^1^H and ^13^C NMR) spectra were obtained on a Bruker Ultra Shield plus 400 MHz spectrometer. Chemical shift was relative to tetramethylsilane (TMS) as the internal standard. Resonance patterns were reported with the notation s (singlet), d (double), t (triplet), q (quartet), and m (multiplet). Mass spectra were obtained on a matrix-assisted laser desorption/ionization time of flight mass spectrometry (MALDI-TOF-MS). UV-visible absorption spectra were measured by Shimadzu UV-1750. Steady-state photoluminescence spectra, phosphorescence spectra, and excitation spectra were measured by using HitachiF-4600. The lifetime spectra were carried out on Edinburgh FLSP920 fluorescence spectrophotometer equipped with a xenon arc lamp (Xe900) or microsecond flash-lamp (*μ*F900). Photoluminescence quantum efficiency was collected on a Hamamatsu Absolute PL Quantum Yield Spectrometer C11347 under ambient condition, the fluorescence and phosphorescence quantum efficiency (*Φ*_F_ and *Φ*_P_) were calculated through the following formulas:

(1) $$\begin{array}{c} \Phi_{P}=\Phi_{E}\times \frac{A_{P}}{A_{E}},\\ \Phi_{F}=\Phi_{E}-\Phi_{P}, \end{array}$$

where *Φ*_E_ refers to the measured total emission quantum efficiency, *A*_P_ and *A*_E_ refer to the integral areas of phosphorescence and photoluminescence components in photoluminescence spectra, respectively. During the measurements, the sample was firstly deposited into a quartz cuvette and put into the fluorescence spectrophotometer (HitachiF-4600). Then, the cuvette with the phosphors was carefully fixed and irradiated by UV light with different power for a certain time. After the photoactivation process, the optical signal was collected. Luminescence photos and videos were taken by Cannon EOS 700D single lens digital cameras with a handheld UV lamp on and off. The fluorescent images of crystal were taken by Nikon DS-Ri2 Microscope Camera. The intensity of the UV lamp was measured by PA05-UVAB513-02 UV light meter.

X-ray crystallography was achieved by using a Bruker SMART APEX-II CCD diffractometer with graphite monochromated Mo-K*α* radiation. The structures of PyCz-B and PyCz-N before and after long photoactivation were measured in 100 K where the molecular motions would be restrained by low temperature. In order to exclude the influence of temperature factors in crystal, the crystal was kept in 100 K for over 10 minutes and then the measurement was started. After the first round of measurement, the crystal was photoactivated by a high-power UV lamp (40 mW/cm^2^) for 10 minutes under room temperature and then the structure of crystal was measured as same as the previous round. Based on our observation, after the second round of measurement, two kinds of crystal both still show long lifetime activated UOP thanks for the restriction of molecular motions by low temperature.

### 4.3. Computational Details

All theoretical calculations in this work were based on the measured crystal data of PyCz-B and PyCz-N before and after long photoactivation. The analysis of the independent gradient model (IGM) was carried out by Multiwfn 3.6 [53] and volume was rendered by VMD 1.9.3 [54] based on the selected molecular dimers in the crystal data of PyCz-B and PyCz-N before and after long photoactivation. The isovalue can be set in VMD 1.9.3 to adjust the scale of isosurface. The free volume distributions were calculated using Materials Studio [58] software with 0.2 Å diameter sensor based on the selected crystal cells of PyCz-B and PyCz-N before and after long photoactivation. The single molecular energy was evaluated at B3LYP/6-31G(d) level by Gaussian 09 program based on the one molecular configuration in the crystal data [59].

## Supplementary Materials

Supplementary 1 Table S1: fluorescence lifetimes (*τ*f) of PyCz-B and PyCz-N before and after long photoactivation under ambient conditions. Table S2: ultralong organic phosphorescence lifetimes (*τ*p) of PyCz-B and PyCz-N before and after long photoactivation under ambient conditions. Table S3: ultralong organic phosphorescence lifetimes (*τ*p) of PyCz-B and PyCz-N before and after long photoactivation under nitrogen. Table S4: the fitting results of the photoactivation process of PyCz-B and PyCz-N activated by UV light with different excitation intensity. Table S5: the fitting result of the process of photoactivation and deactivation of PyCz-B and PyCz-N under different atmospheres in Figure S11 and Figure S12. Table S6: the photoluminescent quantum yield (%) of PyCz-N and PyCz-B before and after long photoactivation under ambient conditions. Table S7: structure data of PyCz-N and PyCz-B before and after long photoactivation for 10 minutes at 100 K. Table S8: the intermolecular interactions of PyCz-B before and after long photoactivation. Table S9: the intermolecular interactions of PyCz-N before and after long photoactivation. Table S10: the photophysical parameters of the dynamic UOP phosphors before and after long photoactivation. Table S11. Calculated occupied volume, free volume, and the proportion of free volume in PyCz-B and PyCz-N before and after long photoactivation. Figure S1: 1H NMR spectrum of PyCz in DMSO. Figure S2: 13C NMR spectrum of PyCz in DMSO. Figure S3: MALDI-TOF spectrum of PyCz molecule. Figure S4: photophysical properties of PyCz molecule in dilute 2-methyltetrahydrofuran (1 × 10^−5^ M). Figure S5: excitation spectra monitoring the UOP emission band at 544 nm for PyCz-B and PyCz-N before and after long photoactivation. Figure S6: lifetime decay curves of the fluorescence emission bands of PyCz-B and PyCz-N before and after long photoactivation under ambient conditions. Figure S7: lifetime decay curves of the phosphorescence emission bands of PyCz-B and PyCz-N before and after long photoactivation under ambient conditions. Figure S8: lifetime decay curves of the phosphorescence emission bands of PyCz-B and PyCz-N before and after long photoactivation under nitrogen. Figure S9: the phosphorescence intensity at 544 nm of PyCz-B and PyCz-N after different photoactivation time by a 365 nm lamp (power = 0.67, 0.56, and 0.39 mW/cm2) under ambient conditions. Figure S10: reversible photoactivation and deactivation cycles of PyCz-B and PyCz-N under ambient conditions. Figure S11: the dynamic UOP properties of PyCz crystals under different atmospheres. Figure S12: the deactivated properties of dynamic UOP for PyCz crystal under different atmospheres. Figure S13: phosphorescence spectra of PyCz-N under different temperatures ranging from 173 to 273 K. The corresponding photographs of PyCz-N under daylight, UV on, and UV off at 273, 253, 233, 213, 193, and 77 K. Figure S14: the deactivation process of PyCz-N in 77 K. Figure S15: the angles between triazine and carbazole units of PyCz in optimized S0, S1, and T1 geometry in gas phases. Figure S16: the calculated molecular interactions in dimers of PyCz-B(i), PyCz-B, PyCz-N(i), and PyCz-N(a). Figure S17: free volume region in single crystal cells of PyCz-B and PyCz-N after long photoactivation. Figure S1: the cavities in PyCz-N before and after long photoactivation. Figure S19: the relative intensity (*I*/*I*_0_) of 544 nm UOP of PyCz-N as a function of long photoactivation and deactivation time. Figure S20: the application of PyCz for rewritable timing data storage.

Supplementary 2 Movie S1: the process of long photoactivation of PyCz-B and PyCz-N under the irradiation of a strong UV lamp with the power of 40 mW/cm2.

Supplementary 3 Movie S2: the afterglow of PyCz-N under different temperature.

Supplementary 4 Movie S3: the writing process of the rewritable visual data storage based on PyCz-N phosphor.

Supplementary 5 Movie S4: the erasing process of the rewritable visual data storage based on PyCz-N phosphor.

## References

[1] Sato O. (2016). Dynamic molecular crystals with switchable physical properties. *Nature Chemistry*.

[2] Stuart M. A. C., Huck W. T. S., Genzer J. (2010). Emerging applications of stimuli-responsive polymer materials. *Nature Materials*.

[3] Wang Y., Zhou K., Huang G. (2014). A nanoparticle-based strategy for the imaging of a broad range of tumours by nonlinear amplification of microenvironment signals. *Nature Materials*.

[4] Chen S., Hong Y., Liu Y. (2013). Full-range intracellular pH sensing by an aggregation-induced emission-active two-channel ratiometric fluorogen. *Journal of the American Chemical Society*.

[5] Ji X., Yao Y., Li J., Yan X., Huang F. (2013). A supramolecular cross-linked conjugated polymer network for multiple fluorescent sensing. *Journal of the American Chemical Society*.

[6] Zhu X., Liu R., Li Y. (2014). An AIE-active boron-difluoride complex: multi-stimuli-responsive fluorescence and application in data security protection. *Chemical Communications*.

[7] Hou X., Ke C., Bruns C. J., McGonigal P. R., Pettman R. B., Stoddart J. F. (2015). Tunable solid-state fluorescent materials for supramolecular encryption. *Nature Communications*.

[8] Chung J. W., Yoon S.-J., Lim S.-J., An B.-K., Park S. Y. (2009). Dual-mode switching in highly fluorescent organogels: binary logic gates with optical/thermal inputs. *Angewandte Chemie International Edition*.

[9] Genovese D., Aliprandi A., Prasetyanto E. A. (2016). Mechano‐ and photochromism from bulk to nanoscale: data storage on individual self-assembled ribbons. *Advanced Functional Materials*.

[10] Margulies D., Felder C. E., Melman G., Shanzer A. (2007). A molecular keypad lock: a photochemical device capable of authorizing password entries. *Journal of the American Chemical Society*.

[11] Erbas-Cakmak S., Kolemen S., Sedgwick A. C. (2018). Molecular logic gates: the past, present and future. *Chemical Society Reviews*.

[12] Xu S., Chen R., Zheng C., Huang W. (2016). Excited state modulation for organic afterglow: materials and applications. *Advanced Materials*.

[13] Gan N., Shi H., An Z., Huang W. (2018). Recent advances in polymer-based metal-free room-temperature phosphorescent materials. *Advanced Functional Materials*.

[14] Hirata S. (2017). Recent advances in materials with room-temperature phosphorescence: photophysics for triplet exciton stabilization. *Advanced Optical Materials*.

[15] Kenry, Chen C., Liu B. (2019). Enhancing the performance of pure organic room-temperature phosphorescent luminophores. *Nature Communications*.

[16] He Z., Zhao W., Lam J. W. Y. (2017). White light emission from a single organic molecule with dual phosphorescence at room temperature. *Nature Communications*.

[17] Chai Z., Wang C., Wang J. (2017). Abnormal room temperature phosphorescence of purely organic boron-containing compounds: the relationship between the emissive behaviorand the molecular packing, and the potential related applications. *Chemical Science*.

[18] Shoji Y., Ikabata Y., Wang Q. (2017). Unveiling a new aspect of simple arylboronic esters: long-lived room-temperature phosphorescence from heavy-atom-free molecules. *Journal of the American Chemical Society*.

[19] Zhao W., He Z., Lam J. W. Y. (2016). Rational molecular design for achieving persistent and efficient pure organic room-temperature phosphorescence. *Chem*.

[20] Gan N., Wang X., Ma H. (2019). Manipulating the stacking of triplet chromophores in the crystal form for ultralong organic phosphorescence. *Angewandte Chemie International Edition*.

[21] He Z., Gao H., Zhang S. (2019). Achieving persistent, efficient, and robust room-temperature phosphorescence from pure organics for versatile applications. *Advanced Materials*.

[22] Narushima K., Kiyota Y., Mori T., Hirata S., Vacha M. (2019). Suppressed triplet exciton diffusion due to small orbital overlap as a key design factor for ultralong-lived room-temperature phosphorescence in molecular crystals. *Advanced Materials*.

[23] Ling K., Shi H., Wang H. (2019). Controllable multiemission with ultralong organic phosphorescence in crystal by isomerization. *Advanced Optical Materials*.

[24] An Z., Zheng C., Tao Y. (2015). Stabilizing triplet excited states for ultralong organic phosphorescence. *Nature Materials*.

[25] Cai S., Shi H., Li J. (2017). Visible-light-excited ultralong organic phosphorescence by manipulating intermolecular interactions. *Advanced Materials*.

[26] Cai S., Shi H., Zhang Z. (2018). Hydrogen-bonded organic aromatic frameworks for ultralong phosphorescence by intralayer *π*-*π* interactions. *Angewandte Chemie International Edition*.

[27] Lucenti E., Forni A., Botta C. (2017). Cyclic triimidazole derivatives: intriguing examples of multiple emissions and ultralong phosphorescence at room temperature. *Angewandte Chemie International Edition*.

[28] Gu L., Shi H., Bian L. (2019). Colour-tunable ultra-long organic phosphorescence of a single-component molecular crystal. *Nature Photonics*.

[29] Hirata S., Totani K., Zhang J. (2013). Efficient persistent room temperature phosphorescence in organic amorphous materials under ambient conditions. *Advanced Functional Materials*.

[30] Wei J., Liang B., Duan R. (2016). Induction of strong long-lived room-temperature phosphorescence of *N*-phenyl-2-naphthylamine molecules by confinement in a crystalline dibromobiphenyl matrix. *Angewandte Chemie International Edition*.

[31] Bolton O., Lee K., Kim H. J., Lin K. Y., Kim J. (2011). Activating efficient phosphorescence from purely organic materials by crystal design. *Nature Chemistry*.

[32] Lee D., Bolton O., Kim B. C., Youk J. H., Takayama S., Kim J. (2013). Room temperature phosphorescence of metal-free organic materials in amorphous polymer matrices. *Journal of the American Chemical Society*.

[33] Bian L., Shi H., Wang X. (2018). Simultaneously enhancing efficiency and lifetime of ultralong organic phosphorescence materials by molecular self-assembly. *Journal of the American Chemical Society*.

[34] Cheng Z., Shi H., Ma H. (2018). Ultralong phosphorescence from organic ionic crystals under ambient conditions. *Angewandte Chemie International Edition*.

[35] Yang Z., Mao Z., Zhang X. (2016). Intermolecular electronic coupling of organic units for efficient persistent room-temperature phosphorescence. *Angewandte Chemie International Edition*.

[36] Hu H., Meier F., Zhao D. (2018). Efficient room-temperature phosphorescence from organic-inorganic hybrid perovskites by molecular engineering. *Advanced Materials*.

[37] Li Q., Zhou M., Yang M., Yang Q., Zhang Z., Shi J. (2018). Induction of long-lived room temperature phosphorescence of carbon dots by water in hydrogen-bonded matrices. *Nature Communications*.

[38] Chen X., Xu C., Wang T. (2016). Versatile room-temperature-phosphorescent materials prepared from N-substituted naphthalimides: emission enhancement and chemical conjugation. *Angewandte Chemie International Edition*.

[39] Yang X., Yan D. (2016). Long-afterglow metal–organic frameworks: reversible guest-induced phosphorescence tunability. *Chemical Science*.

[40] Tao Y., Chen R., Li H. (2018). Resonance-activated spin-flipping for efficient organic ultralong room-temperature phosphorescence. *Advanced Materials*.

[41] Xiao L., Wu Y., Yu Z. (2018). Room-temperature phosphorescence in pure organic materials: halogen bonding switching effects. *Chemistry - A European Journal*.

[42] Kwon M. S., Yu Y., Coburn C. (2015). Suppressing molecular motions for enhanced room-temperature phosphorescence of metal-free organic materials. *Nature Communications*.

[43] Cai S., Ma H., Shi H. (2019). Enabling long-lived organic room temperature phosphorescence in polymers by subunit interlocking. *Nature Communications*.

[44] Wang H., Shi H., Ye W. (2019). Amorphous ionic polymers with color-tunable ultralong organic phosphorescence. *Angewandte Chemie International Edition*.

[45] Gu L., Shi H., Gu M. (2018). Dynamic ultralong organic phosphorescence by photoactivation. *Angewandte Chemie International Edition*.

[46] Yang J., Zhen X., Wang B. (2018). The influence of the molecular packing on the room temperature phosphorescence of purely organic luminogens. *Nature Communications*.

[47] Jia X., Shao C., Bai X. (2019). Photoexcitation-controlled self-recoverable molecular aggregation for flicker phosphorescence. *Proceedings of the National Academy of Sciences of the United States of America*.

[48] Huang Q., Mei X., Xie Z. (2019). Photo-induced phosphorescence and mechanoluminescence switching in a simple purely organic molecule. *Journal of Materials Chemistry C*.

[49] Yan D., Evans D. G. (2014). Molecular crystalline materials with tunable luminescent properties: from polymorphs to multi-component solids. *Materials Horizons*.

[50] Xie Y., Ge Y., Peng Q., Li C., Li Q., Li Z. (2017). How the molecular packing affects the room temperature phosphorescence in pure organic compounds: ingenious molecular design, detailed crystal analysis, and rational theoretical calculations. *Advanced Materials*.

[51] Yang J., Ren Z., Chen B. (2017). Three polymorphs of one luminogen: how the molecular packing affects the RTP and AIE properties?. *Journal of Materials Chemistry C*.

[52] Wang J., Wang C., Gong Y. (2018). Bromine-substituted fluorene: molecular structure, Br–Br interactions, room-temperature phosphorescence, and tricolor triboluminescence. *Angewandte Chemie International Edition*.

[53] Lu T., Chen F. (2012). Multiwfn: a multifunctional wavefunction analyzer. *Journal of Computational Chemistry*.

[54] Humphrey W., Dalke A., Schulten K. (1996). VMD: visual molecular dynamics. *Journal of Molecular Graphics & Modelling*.

[55] De Silva A. P. (2013). *Molecular Logic-Based Computation*.

[56] Zhang K. Y., Yu Q., Wei H., Liu S., Zhao Q., Huang W. (2018). Long-lived emissive probes for time-resolved photoluminescence bioimaging and biosensing. *Chemical Reviews*.

[57] Sun H., Liu S., Lin W. (2014). Smart responsive phosphorescent materials for data recording and security protection. *Nature Communications*.

[58] (2013). *Material Studio, version 7.0*.

[59] Frisch M. J., Trucks G. W., Schlegel H. B. (2009). *Gaussian 09. Revision C.01.*.

