# Supplementary material for: Polymorphism-Dependent Dynamic Ultralong Organic Phosphorescence
Source: Research (Wash D C). 2020 Feb 7;2020:8183450. doi: 10.34133/2020/8183450 (PMC7029214; doi:10.34133/2020/8183450)
Supplement: Supplementary 1 — Table S1: fluorescence lifetimes (τf) of PyCz-B and PyCz-N before and after long photoactivation under ambient conditions. Table S2: ultralong organic phosphorescence lifetimes (τp) of PyCz-B and PyCz-N before and after long photoactivation under ambient conditions. Table S3: ultralong organic phosphorescence lifetimes (τp) of PyCz-B and PyCz-N before and after long photoactivation under nitrogen. Table S4: the fitting results of the photoactivation process of PyCz-B and PyCz-N activated by UV light with different excitation intensity. Table S5: the fitting result of the process of photoactivation and deactivation of PyCz-B and PyCz-N under different atmospheres in Figure S11 and Figure S12. Table S6: the photoluminescent quantum yield (%) of PyCz-N and PyCz-B before and after long photoactivation under ambient conditions. Table S7: structure data of PyCz-N and PyCz-B before and after long photoactivation for 10 minutes at 100 K. Table S8: the intermolecular interactions of PyCz-B before and after long photoactivation. Table S9: the intermolecular interactions of PyCz-N before and after long photoactivation. Table S10: the photophysical parameters of the dynamic UOP phosphors before and after long photoactivation. Table S11. Calculated occupied volume, free volume, and the proportion of free volume in PyCz-B and PyCz-N before and after long photoactivation. Figure S1: 1H NMR spectrum of PyCz in DMSO. Figure S2: 13C NMR spectrum of PyCz in DMSO. Figure S3: MALDI-TOF spectrum of PyCz molecule. Figure S4: photophysical properties of PyCz molecule in dilute 2-methyltetrahydrofuran (1 × 10^−5^ M). Figure S5: excitation spectra monitoring the UOP emission band at 544 nm for PyCz-B and PyCz-N before and after long photoactivation. Figure S6: lifetime decay curves of the fluorescence emission bands of PyCz-B and PyCz-N before and after long photoactivation under ambient conditions. Figure S7: lifetime decay curves of the phosphorescence emission bands of PyCz-B and PyCz-N bef [file 8183450.f1.docx]

Supplemental Information

Contents

1. Experimental section

2. Additional photophysical properties of PyCz in solution

3. Data on dynamic UOP of PyCz crystal.

4. Single crystal analyses

5. Theoretical calculations

6. Application

7. Supporting videos

8. References

1. Experimental section

**Material synthesis**

The material that referred in this article is 2,3-di(9H-carbazol-9-yl) pyridine (PyCz), and it was synthesized through one simple step reaction according to the previous literature [1]. ^1^H NMR (400 MHz, DMSO) δ 8.89 (dd, *J* = 4.6, 1.2 Hz, 1H), 8.41 (d, *J* = 8.0 Hz, 1H), 8.12 - 7.72 (m, 5H), 7.34 - 7.25 (m, 2H), 7.22 (dd, *J* = 6.1, 3.0 Hz, 2H), 7.14 - 6.98 (m, 8H). ^13^C NMR (101 MHz, DMSO) δ 149.57, 146.90, 140.17, 139.55, 139.28, 129.63, 126.31, 126.04, 125.23, 123.51, 123.47, 120.91, 120.81, 120.76, 120.51, 111.03, 109.92. MALDI-TOF (m/z): calcd for C_29_H_19_N_3_, 409.49. Found: 408.773. Anal. Calcd for C_29_H_19_N_3_: C, 85.06%; H, 4.68%; N, 10.26%. Found: C, 84.57%, H, 4.88%, N, 9.85%.

**
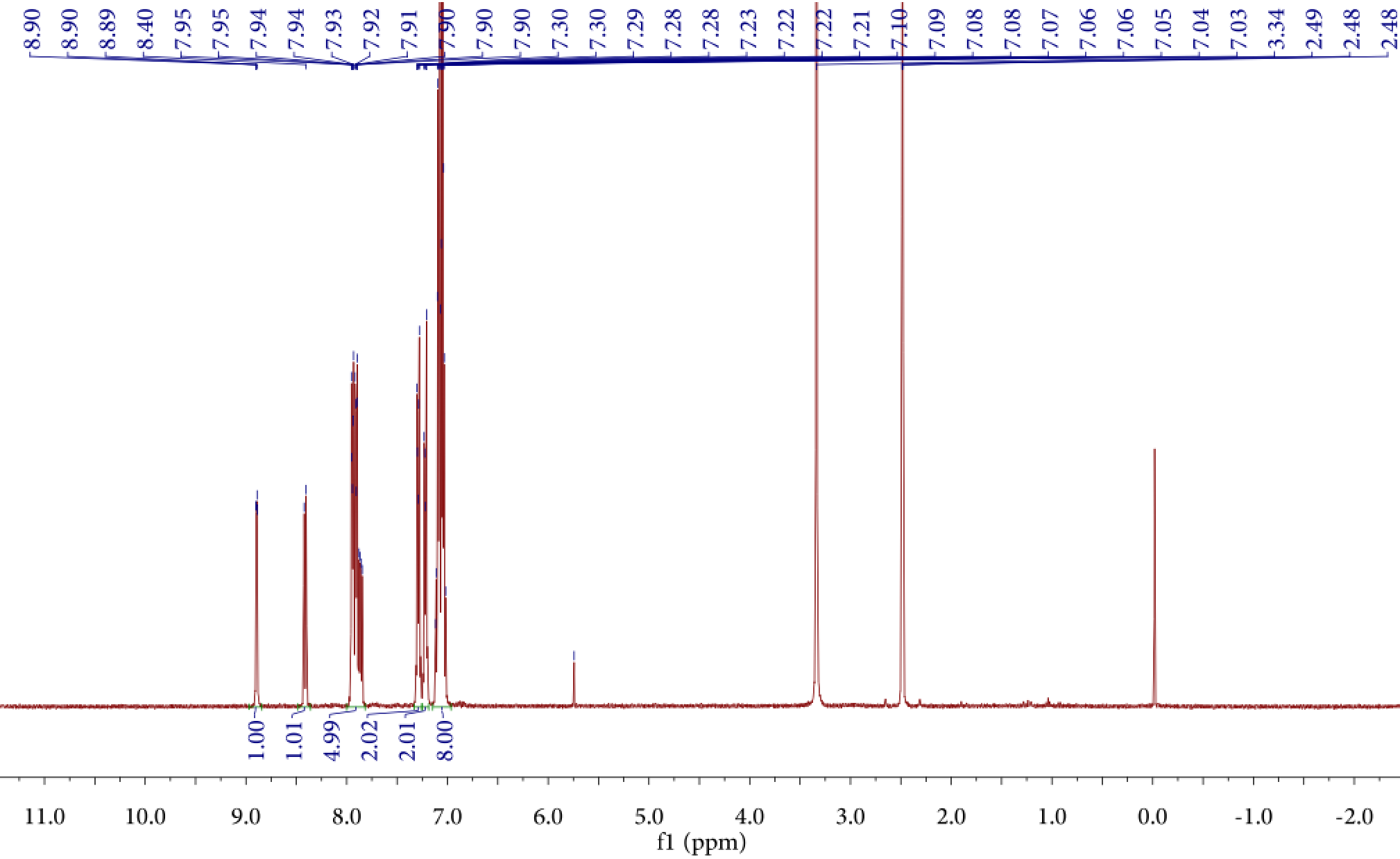
**

**Figure S1.** ^1^H NMR spectrum of PyCz in DMSO.

**
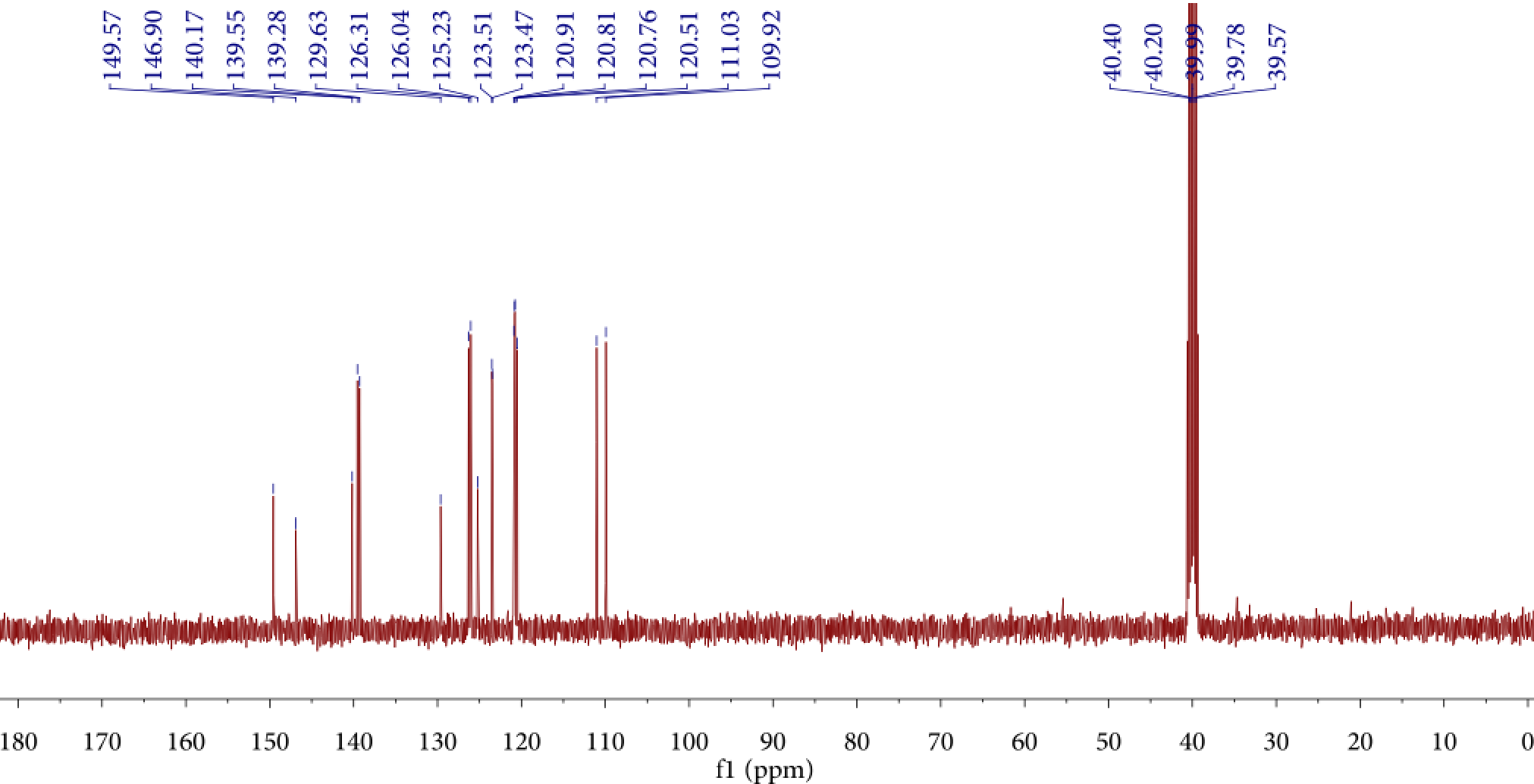
**

**Figure S2.** ^13^C NMR spectrum of PyCz in DMSO.

**
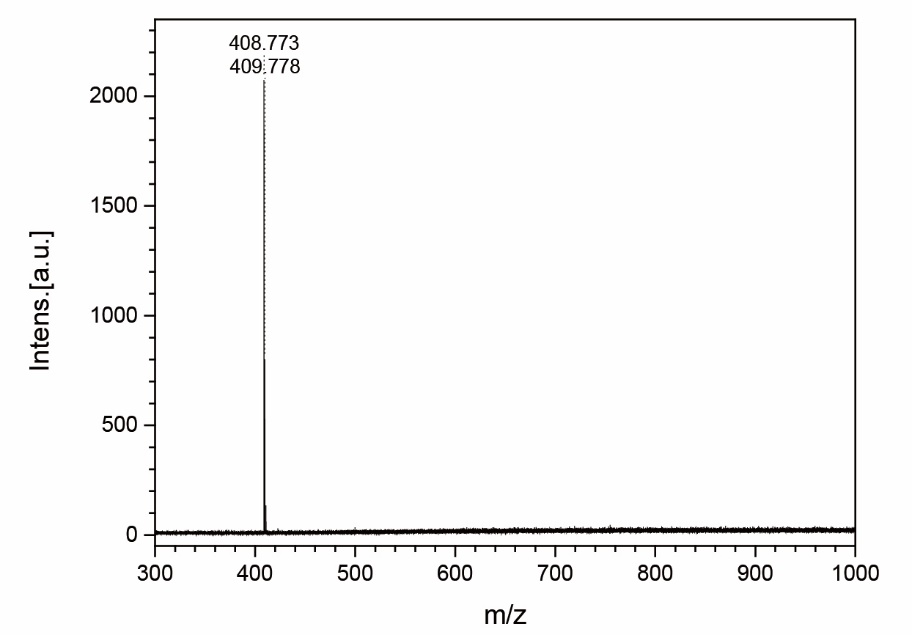
**

**Figure S3.** MALDI-TOF spectrum of PyCz molecule.

2. Additional photophysical properties of PyCz in solution

Photophysical properties of PyCz molecule were thoroughly studied in dilute solution (1x10^-5^ M in 2-methyltetrahydrofuran) through absorption and photoluminescence (PL) spectra. The strong absorption band around 250 nm was ascribed to the pyridine group, and the absorption bands around 290 and 330 nm were ascribed to the carbazole groups [1]. In ambient conditions, the molecule can be excited by UV light and show blue fluorescence emission at around 380 nm. Additionally, due to the suppression of molecular motions at low temperature, the single molecule of PyCz can emit long lifetime phosphorescence under 77 K, whose peaks were at 413, 442 and 463 nm.

**
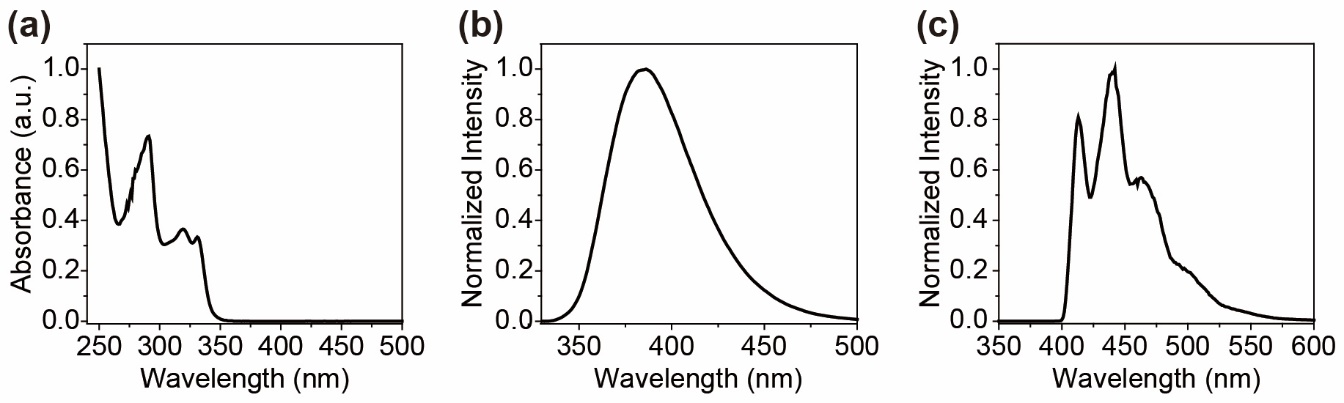
**

**Figure S4.** Photophysical properties of PyCz molecule in dilute 2-methyltetrahydrofuran (1×10^-5^ M). (a). Normalized absorption of PyCz solution under ambient conditions. (b). Steady-state PL spectrum of PyCz solution excited at 320 nm under ambient conditions. (c). Phosphorescence spectrum of PyCz solution excited at 320 nm and at 77 K.

3. Data on dynamic UOP of PyCz crystal.

To gain insight into the dynamic UOP properties, the excitation spectra monitoring the emission band at around 544 nm for PyCz-B and PyCz-N were measured before and after long photoactivation. From Figure S5, it was found that the phosphors can be efficiently activated by the light wavelength ranging from 340 to 400 nm [2].

**
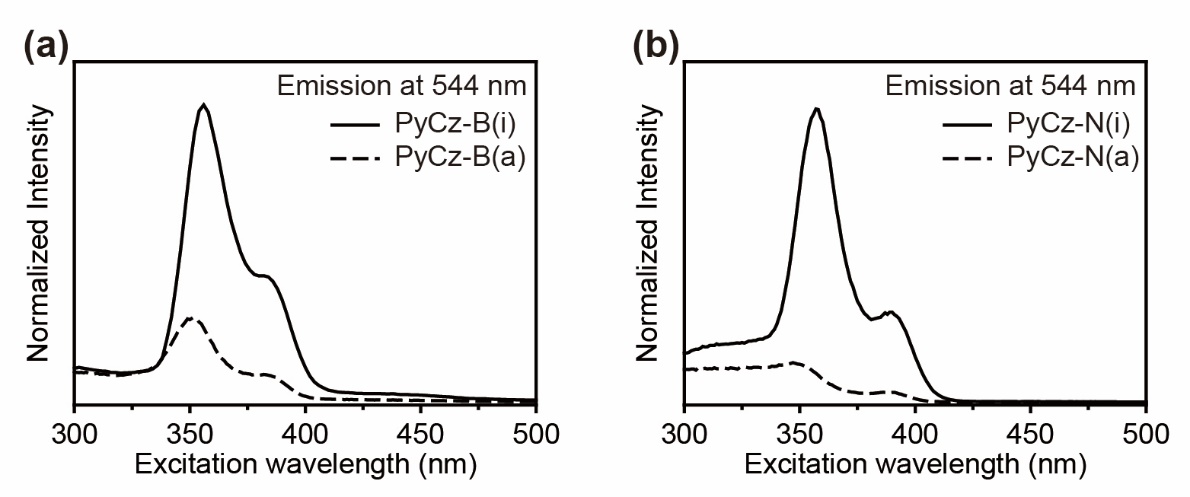
**

**Figure S5.** Excitation spectra monitoring the UOP emission band at 544 nm for PyCz-B (a) and PyCz-N (b) before and after long photoactivation.

**
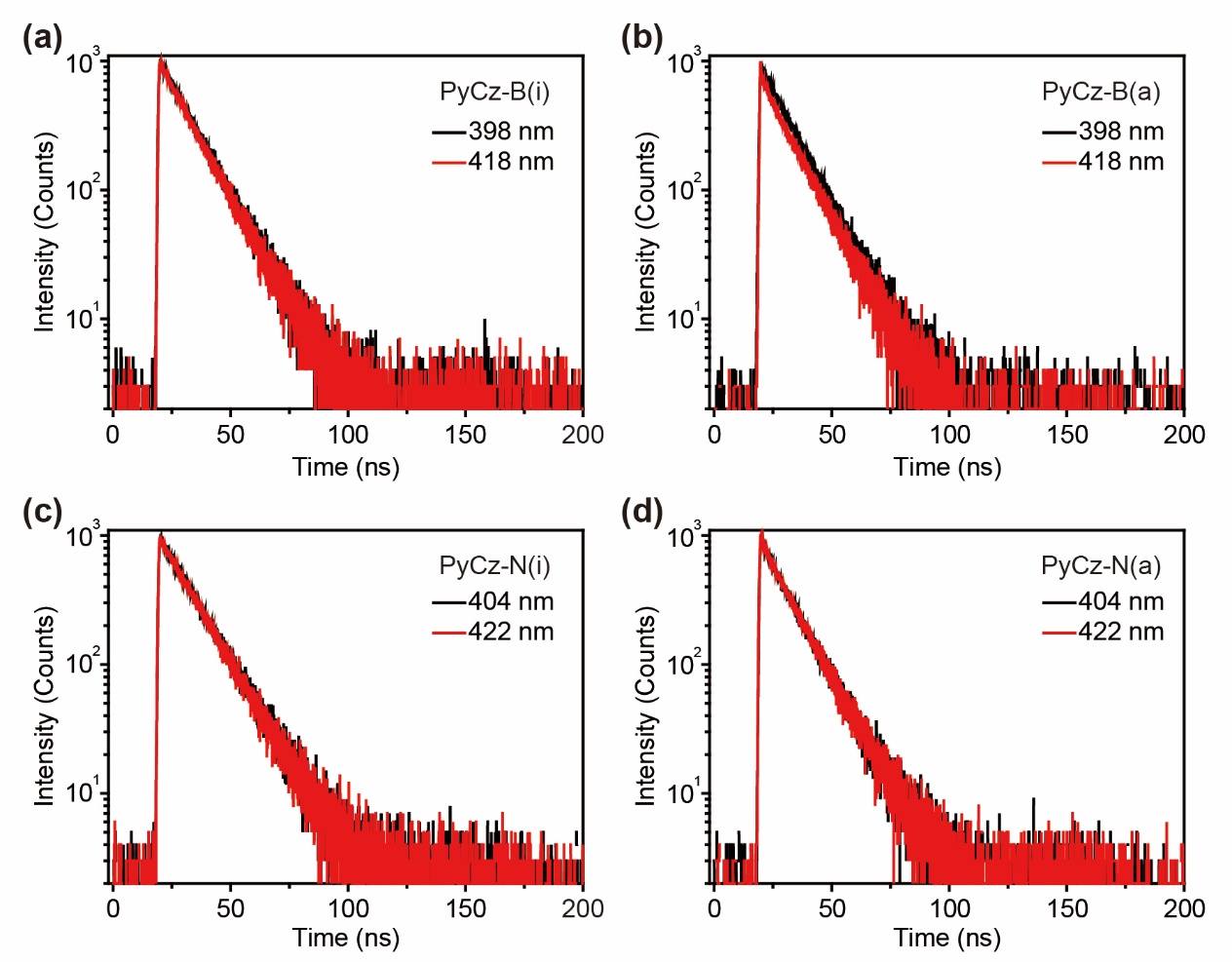
**

**Figure S6.** Lifetime decay curves of the fluorescence emission bands of PyCz-B before (a) and after (b) long photoactivation under ambient conditions. Lifetime decay curves of the fluorescence emission bands of PyCz-N before (c) and after (d) long photoactivation under ambient conditions.

**Table S1.** Fluorescence lifetimes (τ_f_) of PyCz-B and PyCz-N before and after long photoactivation under ambient conditions ^a^.

| Compounds | Wavelength (nm) | Lifetime | |
| --- | --- | --- | --- |
|  |  | τ(ns) | A (%) |
| PyCz-B(i) | 398 | 12.84 | 100 |
|  | 418 | 12.57 | 100 |
| PyCz-B(a) | 398 | 12.45 | 100 |
|  | 418 | 12.14 | 100 |
| PyCz-N(i) | 404 | 13.78 | 100 |
|  | 422 | 13.66 | 100 |
| PyCz-N(a) | 404 | 12.48 | 100 |
|  | 422 | 12.34 | 100 |

^a^ Determined from the fitting function of I(t) = A e^-t/τ^ according to the fluorescence decay curves.

**
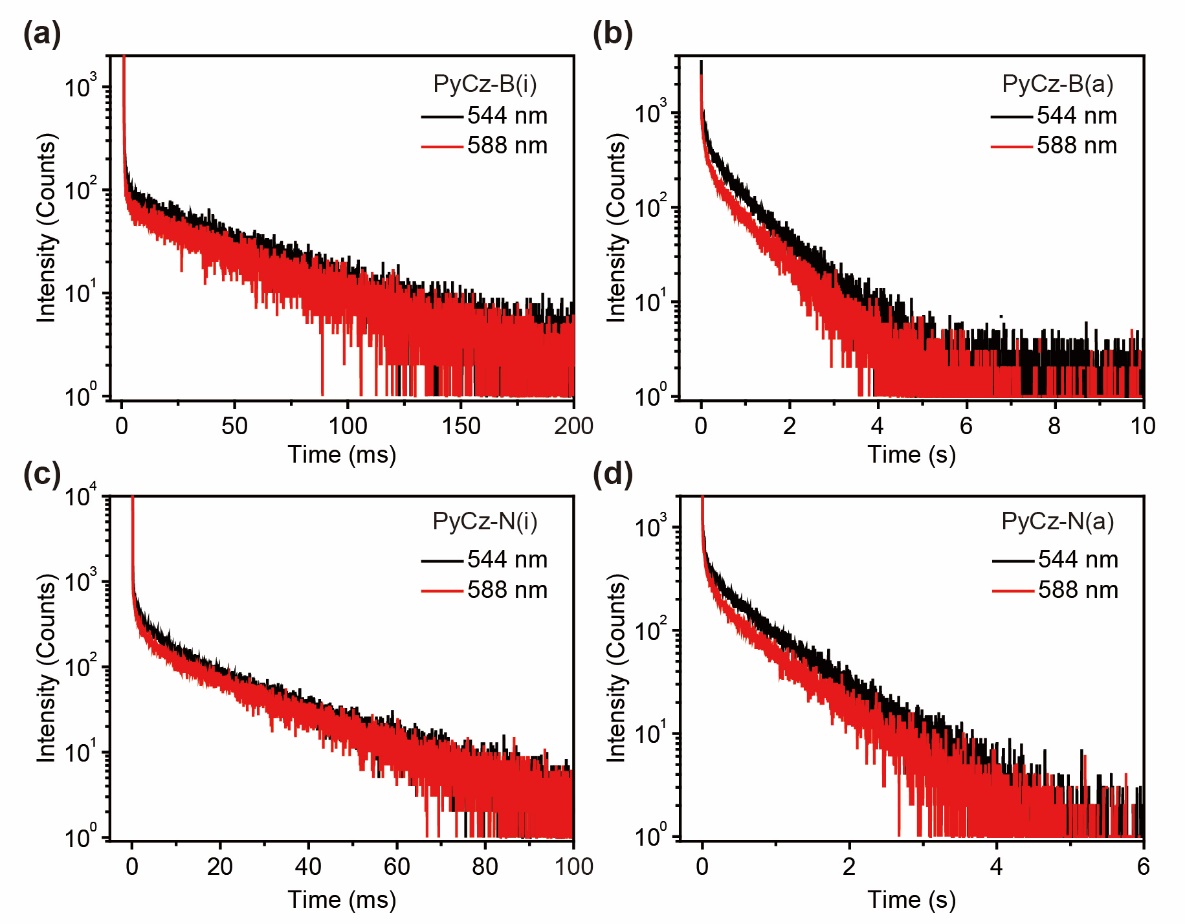
**

**Figure S7.** Lifetime decay curves of the phosphorescence emission bands of PyCz-B before (a) and after (b) long photoactivation under ambient conditions. Lifetime decay curves of the phosphorescence emission bands of PyCz-N before (c) and after (d) long photoactivation under ambient conditions.

**Table S2.** Ultralong organic phosphorescence lifetimes (τ_p_) of PyCz-B and PyCz-N before and after long photoactivation under ambient conditions ^a^.

| Compounds | Wavelength (nm) | Lifetime | | | |
| --- | --- | --- | --- | --- | --- |
|  |  | τ_1_ (ms) | A_1_ (%) | τ_2_ (ms) | A_2_ (%) |
| PyCz-B(i) | 544 | 44.52 | 100.00 | - | - |
|  | 588 | 1.84 | 2.27 | 46.19 | 97.73 |
| PyCz-B(a) | 544 | 87.33 | 15.37 | 868.86 | 84.63 |
|  | 588 | 79.26 | 18.44 | 822.00 | 81.56 |
| PyCz-N(i) | 544 | 2.91 | 18.06 | 20.24 | 81.94 |
|  | 588 | 2.50 | 12.62 | 20.86 | 87.38 |
| PyCz-N(a) | 544 | 49.73 | 10.36 | 776.03 | 89.64 |
|  | 588 | 61.06 | 15.37 | 729.97 | 84.63 |

^a^ Determined from the fitting function of I(t) = A_1_ e^-t/τ1^ + A_2_ e^-t/τ2^ according to the ultralong luminescence decay curves.

**
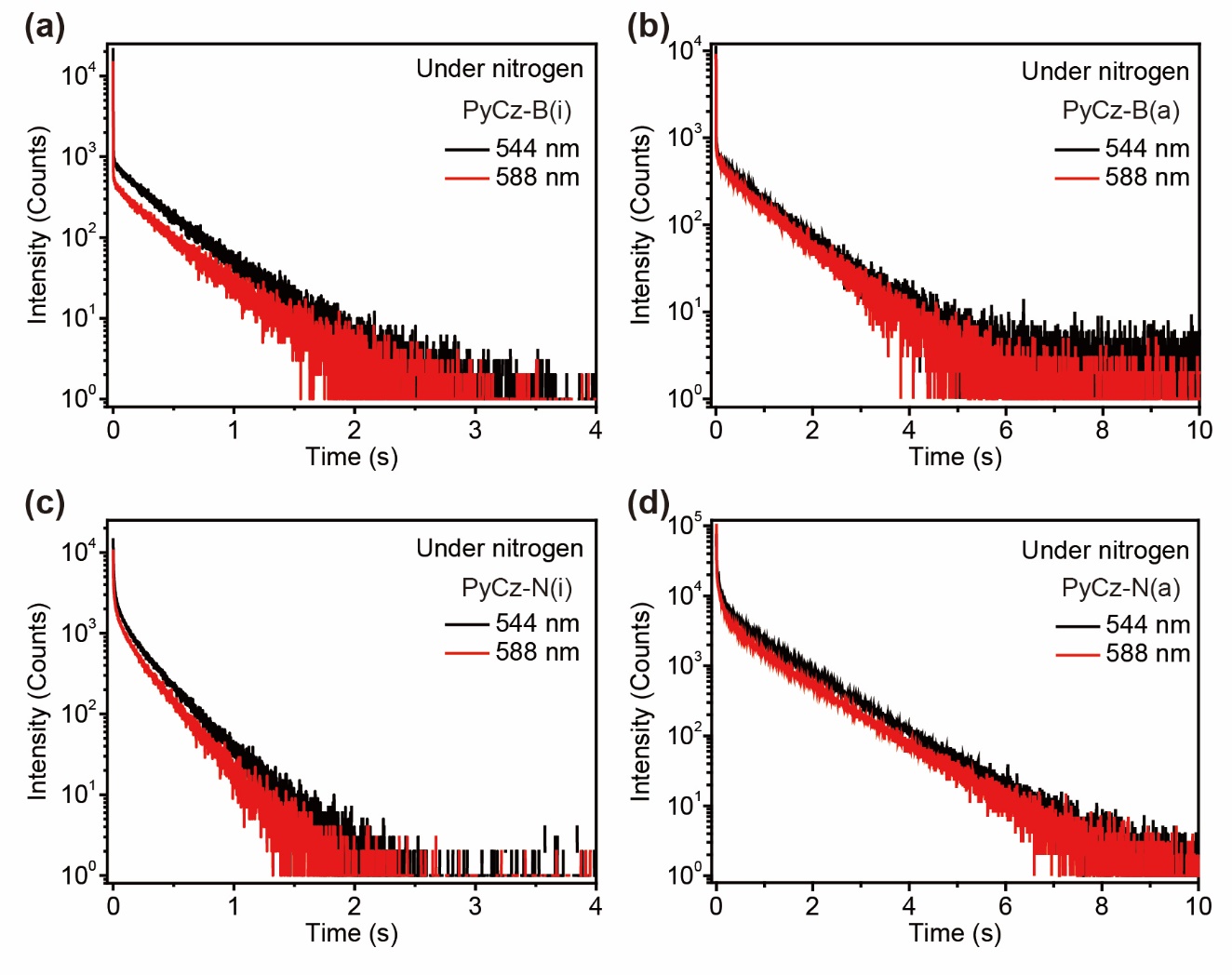
**

**Figure S8.** Lifetime decay curves of the phosphorescence emission bands of PyCz-B before (a) and after (b) long photoactivation under nitrogen. Lifetime decay curves of the phosphorescence emission bands of PyCz-N before (c) and after (d) long photoactivation under nitrogen.

**Table S3.** Ultralong organic phosphorescence lifetimes (τ_p_) of PyCz-B and PyCz-N before and after long photoactivation under nitrogen ^a^.

| Compounds | Wavelength (nm) | Lifetime | | | | | | | |
| --- | --- | --- | --- | --- | --- | --- | --- | --- | --- |
|  |  | τ_1_  (ms) | A_1_  (%) | τ_2_  (ms) | A_2_  (%) | τ_3_  (ms) | A_3_  (%) | τ_4_  (ms) | A_4_  (%) |
| PyCz-B(i) | 544 | 115.34 | 11.42 | 397.78 | 88.58 |  |  |  |  |
|  | 588 | 56.92 | 5.80 | 364.19 | 94.20 |  |  |  |  |
| PyCz-B(a) | 544 | 165.23 | 5.84 | 965.96 | 94.16 |  |  |  |  |
|  | 588 | 150.23 | 6.04 | 949.60 | 93.96 |  |  |  |  |
| PyCz-N(i) | 544 | 4.15 | 5.39 | 46.76 | 19.16 | 264.13 | 75.46 |  |  |
|  | 588 | 3.34 | 4.24 | 46.87 | 18.48 | 234.98 | 77.29 |  |  |
| PyCz-N(a) | 544 | 42.78 | 5.19 | 117.54 | 10.08 | 456.26 | 17.09 | 1065.46 | 67.65 |
|  | 588 | 2.28 | 2.68 | 56.72 | 13.54 | 286.89 | 18.24 | 1031.58 | 65.54 |

^a^ Determined from the fitting function of I(t) = A_1_ e^-t/τ1^ + A_2_ e^-t/τ2^ + A_3_ e^-t/τ3^ + A_4_ e^-t/τ4^according to the ultralong luminescence decay curves.


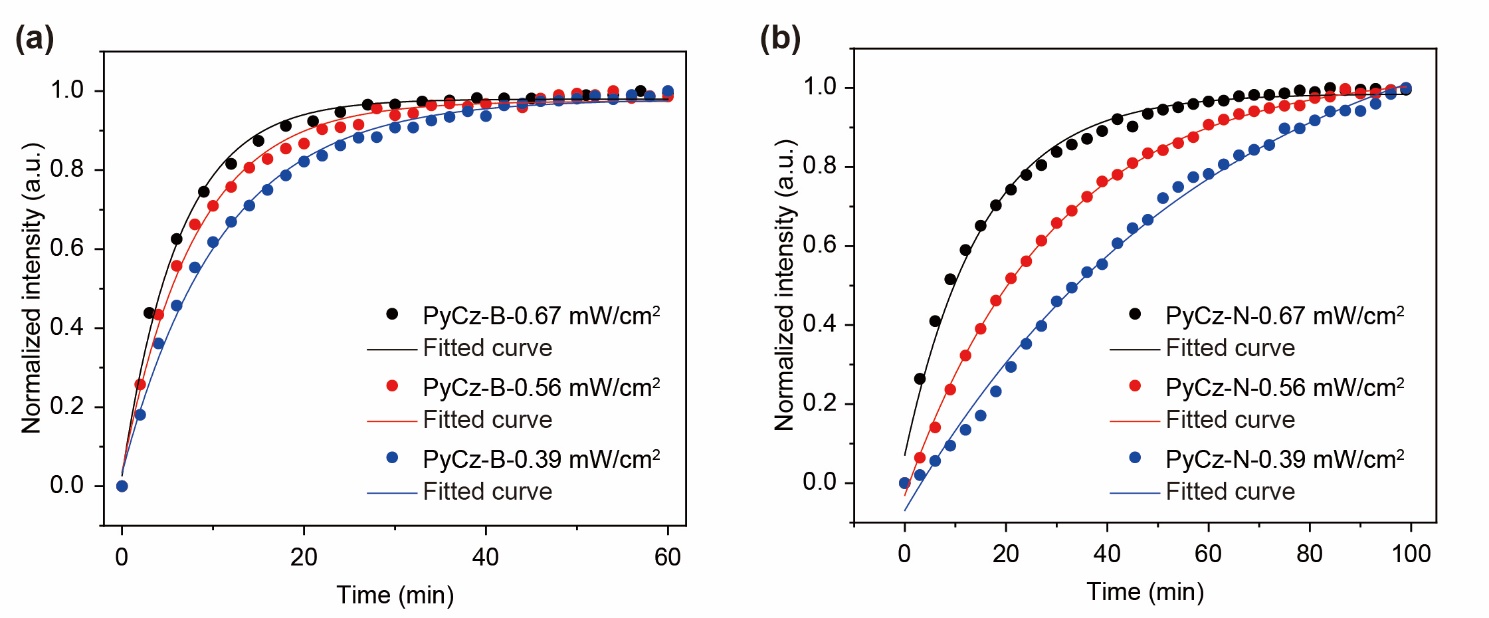


**Figure S9.** The corresponding peak intensity at 544 nm of PyCz-B (a) and PyCz-N (b) after different photoactivation time by a 365 nm lamp (power = 0.67, 0.56 and 0.39 mW/cm2) under ambient conditions.

**Table S4.** The fitting results of the photoactivation process of PyCz-B and PyCz-N activated by UV light with different excitation intensity.

|  | PyCz-N | | | PyCz-B | | |
| --- | --- | --- | --- | --- | --- | --- |
|  | 0.67 mW/cm^2^ | 0.56 mW/cm^2^ | 0.39 mW/cm^2^ | 0.67 mW/cm^2^ | 0.56 mW/cm^2^ | 0.39 mW/cm^2^ |
| Equation | $I_{t}=\Delta Ie^{-kt}+I_{\infty}$ | | | | | |
| $I_{\infty}$ | 0.98 | 0.97 | 0.98 | 0.99 | 1.04 | 1.28 |
| Std. Dev. | 3.22×10^-3^ | 4.84×10^-3^ | 5.49×10^-3^ | 6.08×10^-3^ | 5.07×10^-3^ | 4.33×10^-2^ |
| $\Delta I$ | -0.95 | -0.94 | -0.95 | -0.91 | -1.07 | -1.35 |
| Std. Dev. | 15.04×10^-3^ | 1.51×10^-2^ | 1.27×10^-2^ | 16.77×10^-3^ | 6.13×10^-3^ | 3.73×10^-2^ |
| k | 15.57×10^-2^ | 12.58×10^-2^ | 9.17×10^-2^ | 6.50×10^-2^ | 3.37×10^-2^ | 1.62×10^-2^ |
| Std. Dev. | 4.51×10^-3^ | 3.83×10^-3^ | 2.59×10^-3^ | 2.32×10^-3^ | 5.42×10^-4^ | 9.92×10^-4^ |
| τ | 6.42 | 7.95 | 10.90 | 15.39 | 29.66 | 61.68 |
| R^2^ | 0.9938 | 0.9940 | 0.9952 | 0.9913 | 0.9990 | 0.9948 |

**
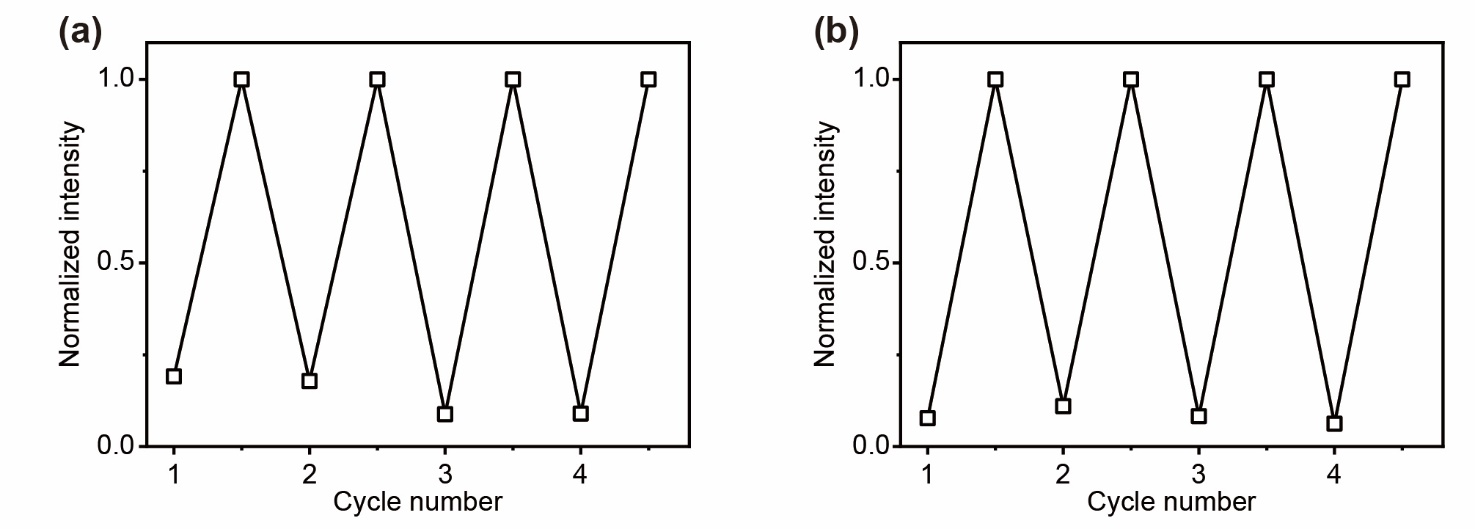
**

**Figure S10.** Reversible photoactivation and deactivation cycles of PyCz-B (a) and PyCz-N (b) under ambient conditions.

The process of photoactivation and deactivation of ultralong organic phosphorescence of PyCz-B and PyCz-N were subject to the first order kinetic model, and the relation between dynamic phosphorescence intensity (I_t_) and time (t) can be fitted by using the first-order kinetic equation of

$$I_{t}=\Delta Ie^{-kt}+I_{\infty}$$

where $I_{\infty}$ refers to the limit value of phosphorescence intensity (t=∞). $\Delta I$ represents the changed value between initial phosphorescence intensity ($I_{0}$) and $I_{\infty}$ during the dynamic process, that is $\Delta I=I_{0}-I_{\infty}$. k is the rate constant of photoactivation and deactivation. Here, we defined τ=1/k as the photoactivation or deactivation lifetime, it refers to the needed time for the dynamic phosphorescence intensity changing to 1/e of $\Delta I$ during the dynamic process.

**
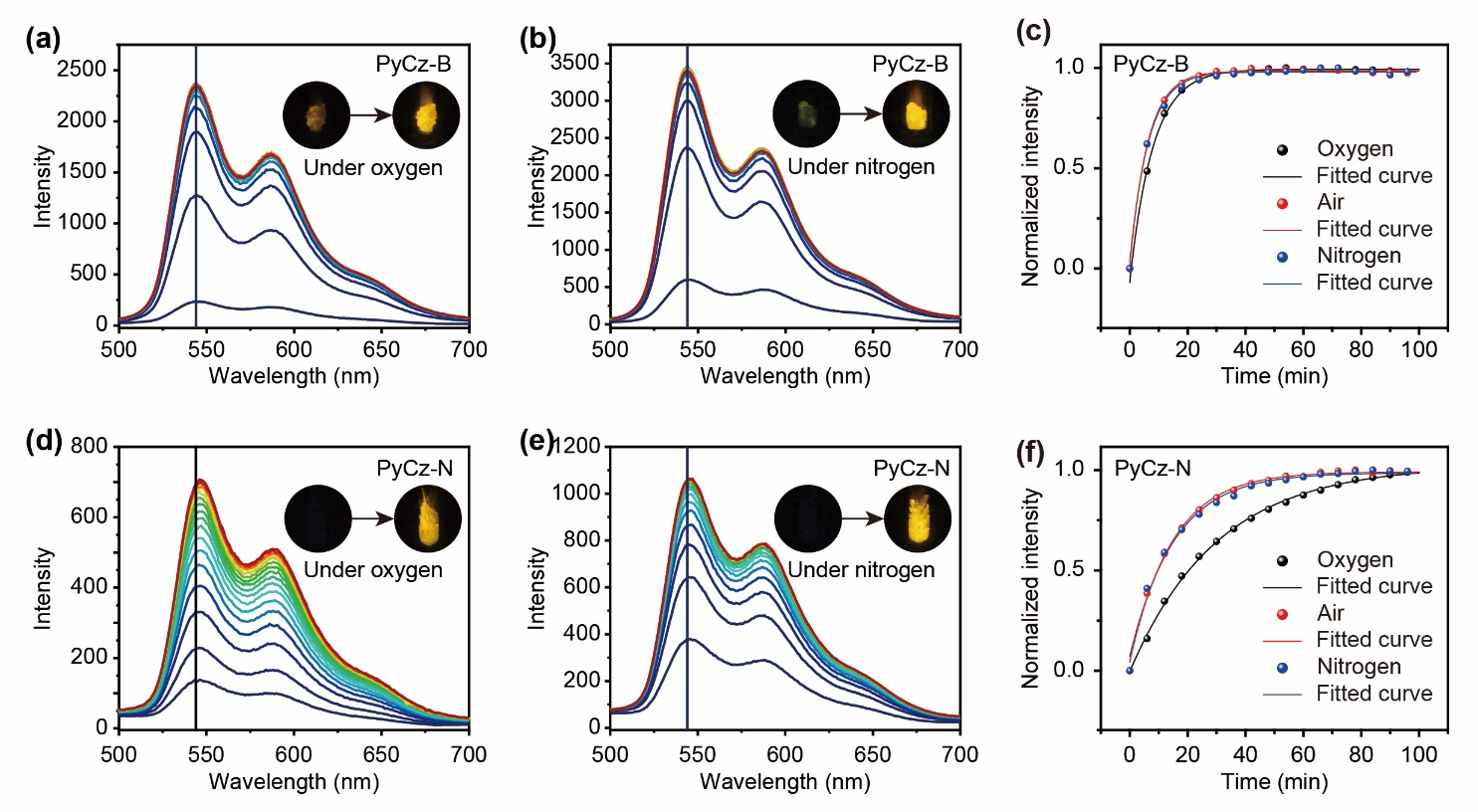
**

**Figure S11.** The dynamic UOP properties of PyCz crystals under different atmosphere. Phosphorescence spectra of PyCz-B and PyCz-N crystal after different photoactivated time (0 to 100 min) under oxygen (a, d) and nitrogen (b, e) by weak UV light with power of 0.67 mW/cm^2^ as well as the peak intensity (544 nm) and activation time (c, f), respectively.

**
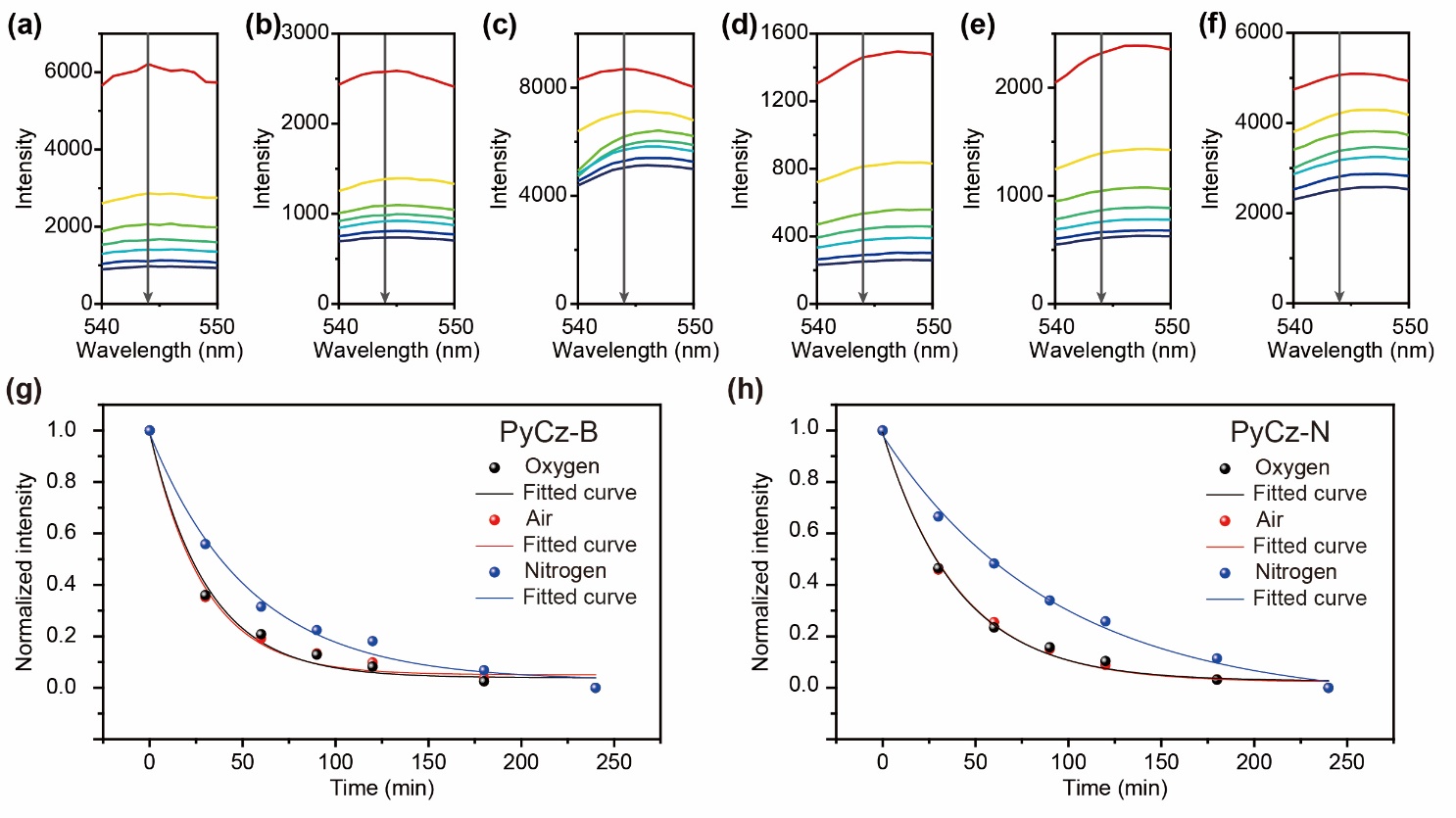
**

**Figure S12.** The deactivated properties of dynamic UOP for PyCz crystal under different atmosphere. The variation of UOP intensity during deactivation process for PyCz-B and PyCz-N under oxygen (a, d), air (b, e) and nitrogen (c, f) within 240 min. The peak intensity (544 nm) and deactivation time of PyCz-B (g) and PyCz-N (h).

**Table S5.** The fitting result of the process of photoactivation and deactivation of PyCz-B and PyCz-N under different atmosphere in Figure S11 and Figure S12.

|  | | PyCz-N | | | PyCz-B | | |
| --- | --- | --- | --- | --- | --- | --- | --- |
|  |  | Oxygen | Air | Nitrogen | Oxygen | Air | Nitrogen |
| Photoactivation | Equation | $I_{t}=\Delta Ie^{-kt}+I_{\infty}$ | | | | | |
|  | $I_{\infty}$ | 1.02 | 0.99 | 0.99 | 0.99 | 0.98 | 0.98 |
|  | Std. Dev. | 4.55×10^-3^ | 6.08×10^-3^ | 2.42×10^-3^ | 3.13×10^-3^ | 3.22×10^-3^ | 1.11×10^-3^ |
|  | $\Delta I$ | -1.03 | -0.91 | -0.95 | -1.06 | -0.95 | -0.98 |
|  | Std. Dev. | 5.43×10^-3^ | 16.77×10^-3^ | 7.41×10^-3^ | 14.75×10^-3^ | 15.04×10^-3^ | 5.86×10^-3^ |
|  | k | 3.30×10^-2^ | 6.50×10^-2^ | 6.84×10^-2^ | 12.51×10^-2^ | 15.57×10^-2^ | 16.11×10^-2^ |
|  | Std. Dev. | 0.50×10^-3^ | 2.32×10^-3^ | 1.02×10^-3^ | 2.99×10^-3^ | 4.51×10^-3^ | 1.69×10^-3^ |
|  | τ | 30.27 | 15.39 | 14.63 | 7.99 | 6.42 | 6.21 |
|  | R^2^ | 0.9987 | 0.9913 | 0.9977 | 0.9932 | 0.9938 | 0.9987 |
| Deactivation | Equation | $I_{t}=\Delta Ie^{-kt}+I_{\infty}$ | | | | | |
|  | $I_{\infty}$ | 2.61×10^-2^ | 2.17×10^-2^ | -5.39×10^-2^ | 3.9×10^-2^ | 5.04×10^-2^ | 2.80×10^-2^ |
|  | Std. Dev. | 1.86×10^-2^ | 1.63×10^-2^ | 4.28×10^-2^ | 2.41×10^-2^ | 2.44×10^-2^ | 3.13×10^-2^ |
|  | $\Delta I$ | 0.97 | 0.97 | 1.04 | 0.95 | 0.94 | 0.96 |
|  | Std. Dev. | 3.10×10^-2^ | 2.68×10^-2^ | 4.23×10^-2^ | 4.53×10^-2^ | 4.72×10^-2^ | 4.36×10^-2^ |
|  | k | 2.48×10^-2^ | 2.44×10^-2^ | 1.07×10^-2^ | 3.20×10^-2^ | 3.42×10^-2^ | 1.86×10^-2^ |
|  | Std. Dev. | 1.80×10^-2^ | 1.54×10^-2^ | 1.02×10^-2^ | 3.41×10^-2^ | 3.86×10^-2^ | 1.88×10^-2^ |
|  | τ | 40.28 | 40.99 | 93.15 | 31.25 | 29.22 | 53.73 |
|  | R^2^ | 0.9960 | 0.9970 | 0.9957 | 0.9910 | 0.9901 | 0.9921 |

To further understand the relationship between molecular motions and photoactivated dynamic UOP for PyCz, PyCz-N was chosen as a model to study. It is well known that non-radiative transitions $K_{nr}^{Phos}$can be effectively restrained at low temperature because of the frozen molecular motions. With temperature decreasing, the phosphorescence intensity of PyCz-N without photoactivation was gradually enhanced along with visual persistent luminescence emerging. (Figure S13 and Movie S2). Moreover, the deactivation process can be obviously prolonged (Figure S14). Based on the results above, it can be reasoned that non-radiative transition from the molecular motions in crystal played a critical role in generating the dynamic UOP for PyCz crystal during the irradiation of UV light.

**
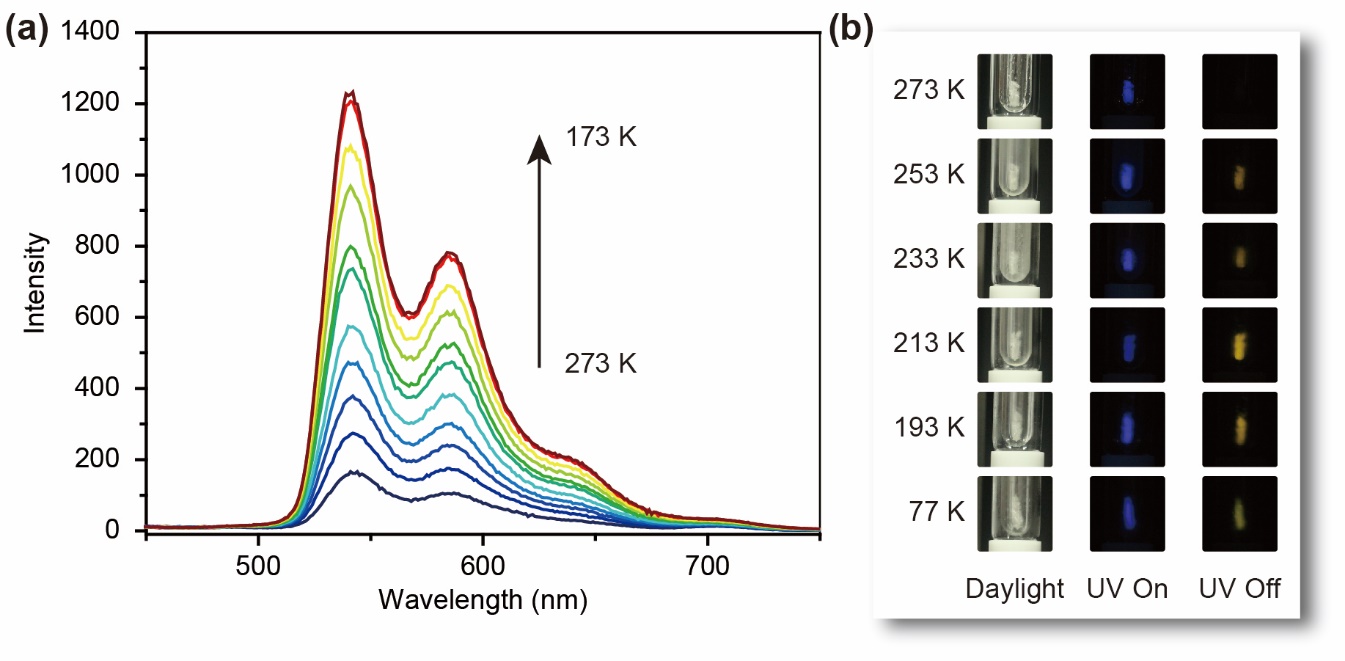
**

**Figure S13.** (a) Phosphorescence spectra of PyCz-N under different temperatures ranging from 173 to 273 K. (b) The corresponding photographs of PyCz-N under daylight, UV on and UV off at 273, 253, 233, 213, 193 and 77 K.

**
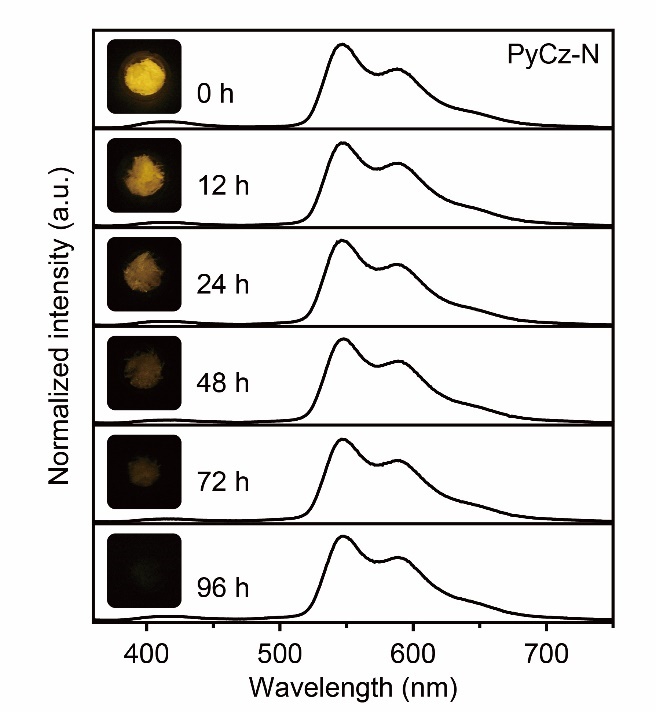
**

**Figure S14.** The deactivation process of PyCz-N in 77 K. The phosphorescence spectra of PyCz-N excited at 350 nm at different periods of time after long photoactivation at 77 K. Insets: corresponding ultralong phosphorescence photographs of PyCz-N.

**Table S6.** The photoluminescent quantum yield (%) of PyCz-N and PyCz-B before and after long photoactivation under ambient conditions.

| Compounds | $\Phi_{F}$/% | $\Phi_{P}$/% |
| --- | --- | --- |
| PyCz-B(i) | 23.22 | 0.18 |
| PyCz-B(a) | 16.20 | 0.80 |
| PyCz-N(i) | 40.91 | 0.09 |
| PyCz-N(a) | 15.77 | 0.23 |

4. Single crystal analyses

**Table S7.** Structure data of PyCz-N and PyCz-B before and after long photoactivation for 10 minutes at 100 K.

|  | PyCz-B(i) | PyCz-B(a) | PyCz-N(i) | PyCz-N(a) |
| --- | --- | --- | --- | --- |
| Formula | C_29_H_19_N_3_ | C_29_H_19_N_3_ | C_29_H_19_N_3_ | C_29_H_19_N_3_ |
| Formula weight | 409.47 | 409.47 | 409.47 | 409.47 |
| Wavelength (Å) | 0.71073 | 0.71073 | 0.71073 | 0.71073 |
| Temperature/K | 100.01 | 99.99 | 100.0 | 100.02 |
| Crystal system | triclinic | triclinic | trigonal | trigonal |
| Space group | P-1 | P-1 | R-3 | R-3 |
| Cell Length (Å) | a 9.402(2)  b 9.992(2)  c 12.883(4) | a 9.399(1)  b 9.979 (1)  c 12.856(2) | a 26.891(8)  b 26.891(8)  c 16.214(5) | a 26.786(10)  b 26.786(10)  c 16.075(6) |
| Cell Angel (°) | α 67.206(4)  β 88.807(7)  γ 77.724(4) | α 67.250(3)  β 81.819(3)  γ 77.770(3) | α 90  β 90  γ 120 | α 90  β 90  γ120 |
| Volume (Å^3)^ | 1087.8(4) | 1084.3(3) | 10154(7) | 9988(8) |
| Z | 2 | 2 | 18 | 18 |
| Density (g/cm^3^) | 1.250 | 1.254 | 1.205 | 1.225 |
| F (000) | 428.0 | 428.0 | 3852.0 | 3852.0 |
| h_max_, k_max_, l_max_ | 16, 16, 20 | 15, 16, 21 | 36, 36, 21 | 37, 36, 22 |
| T_min_, T_max_ | 0.982, 0.989 | 0.982, 0.989 | 0.983, 0.989 | 0.983, 0.989 |
| CCDC number | 1835613 | 1835612 | 1827443 | 1827442 |

**Table S8.** The intermolecular interactions of PyCz-B before and after long photoactivation.

| Number | Interaction type | PyCz-B(i) | PyCz-B(a) | Reduction value |
| --- | --- | --- | --- | --- |
| 1 | C-H···π | 2.814 | 2.810 | 0.004 |
| 2 | C-H···π | 2.768 | 2.764 | 0.004 |
| 3 | C-H···π | 2.849 | 2.843 | 0.006 |
| 4 | C-H···π | 2.732 | 2.730 | 0.002 |
| 5 | C-H···π | 2.845 | 2.841 | 0.004 |
| 6 | C-H···π | 2.762 | 2.753 | 0.009 |
| 7 | C-H···π | 2.843 | 2.836 | 0.007 |
| 8 | C-H···π | 2.819 | 2.816 | 0.003 |
| 9 | C-H···π | - | 2.895 | - |

**Table S9.** The intermolecular interactions of PyCz-N before and after long photoactivation.

| Number | Interaction type | PyCz-N(i) | PyCz-N(a) | Reduction value |
| --- | --- | --- | --- | --- |
| 1 | C-H···π | 2.744 | 2.719 | 0.025 |
| 2 | C-H···π | 2.873 | 2.853 | 0.020 |
| 3 | C-H···π | 2.701 | 2.680 | 0.021 |
| 4 | π···π | 3.400 | 3.377 | 0.023 |
| 5 | π···π | 3.378 | 3.359 | 0.019 |
| 6 | C-H···N | 2.633 | 2.609 | 0.024 |
| 7 | C-H···π | 2.866 | 2.847 | 0.019 |
| 8 | C-H···π | 2.817 | 2.793 | 0.024 |
| 9 | C-H···N | - | 2.748 | - |
| 10 | π···π | - | 3.397 | - |
| 11 | π···π | - | 3.397 | - |
| 12 | π···π | - | 3.399 | - |
| 13 | π···π | - | 3.396 | - |
| 14 | π···π | - | 3.388 | - |

5. Theoretical calculations

**Table S10.** The photophysical parameters of the dynamic UOP phosphors before and after long photoactivation.

| Compounds | Fluorescence | | | | Phosphorescence | | | |
| --- | --- | --- | --- | --- | --- | --- | --- | --- |
|  | $\tau_{F}$  */*ns | $\Phi_{F}$  /% | $k_{r}^{F}$  /10^7^ s^-1^ | $k_{isc}$  /10^7^ s^-1^ | $\tau_{P}$  */*s | $\Phi_{P}$  /% | $k_{r}^{P}$  /10^-2^ s^-1^ | $k_{nr}^{P}$  /s^-1^ |
| PyCz-B(i) | 12.84 | 23.22 | 1.81 | 5.98 | 0.046 | 0.18 | 5.10 | 21.74 |
| PyCz-B(a) | 12.45 | 16.20 | 1.30 | 6.73 | 0.869 | 0.80 | 1.10 | 1.15 |
| PyCz-N(i) | 13.78 | 40.91 | 2.97 | 4.29 | 0.020 | 0.09 | 7.62 | 50.00 |
| PyCz-N(a) | 12.48 | 15.77 | 1.26 | 6.75 | 0.776 | 0.23 | 0.35 | 1.29 |

$\boldsymbol{k}_{\boldsymbol{r}}^{\boldsymbol{F}}\boldsymbol{=}\boldsymbol{\Phi}_{\boldsymbol{F}}\boldsymbol{/}\boldsymbol{\tau}_{\boldsymbol{F}}$***;***$\boldsymbol{\Phi}_{\boldsymbol{isc}}\boldsymbol{=}\boldsymbol{1}\boldsymbol{-}\boldsymbol{\Phi}_{\boldsymbol{F}}\boldsymbol{-}\boldsymbol{\Phi}_{\boldsymbol{ic}}\boldsymbol{\approx}\boldsymbol{1}\boldsymbol{-}\boldsymbol{\Phi}_{\boldsymbol{F}}$***;*** $\boldsymbol{k}_{\boldsymbol{isc}}\boldsymbol{=}\boldsymbol{\Phi}_{\boldsymbol{isc}}\boldsymbol{/}\boldsymbol{\tau}_{\boldsymbol{F}}$***;***$\boldsymbol{k}_{\boldsymbol{nr}}^{\boldsymbol{F}}\boldsymbol{=1/}\boldsymbol{\tau}_{\boldsymbol{F}}\boldsymbol{-}\boldsymbol{k}_{\boldsymbol{r}}^{\boldsymbol{F}}\boldsymbol{-}\boldsymbol{k}_{\boldsymbol{isc}}$***;*** $\boldsymbol{k}_{\boldsymbol{r}}^{\boldsymbol{P}}\boldsymbol{=}\boldsymbol{\Phi}_{\boldsymbol{P}}\boldsymbol{/(}\boldsymbol{\Phi}_{\boldsymbol{isc}}\boldsymbol{\times}\boldsymbol{\tau}_{\boldsymbol{P}}\boldsymbol{)}$***;*** $\boldsymbol{k}_{\boldsymbol{nr}}^{\boldsymbol{P}}\boldsymbol{=1/}\boldsymbol{\tau}_{\boldsymbol{P}}\boldsymbol{-}\boldsymbol{k}_{\boldsymbol{r}}^{\boldsymbol{P}}$

To reveal the driving force of the dynamic phosphorescence from PyCz-B and PyCz-N, we calculated the electronic structures nature of PyCz in gas. When excited from ground state (S_0_) to excited state (S_1_), the molecular configuration of PyCz changes with a larger torsion angle (over 30^o^) between carbazole and pyridine groups. Thus, we suspected the molecular motions may be caused by the changes in molecular configuration between the ground and excited state.

**
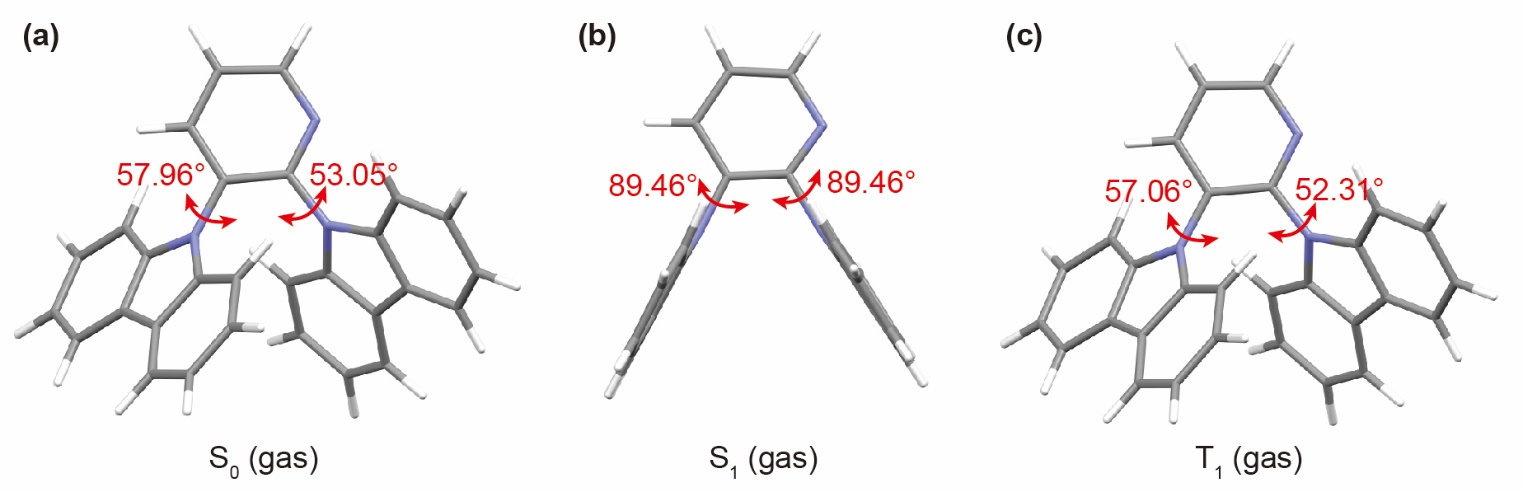
**

**Figure S15.** The angles between triazine and carbazole units of PyCz in optimized S_0_ (a), S_1_ (b) and T_1_ (c) geometry in gas phases.

**
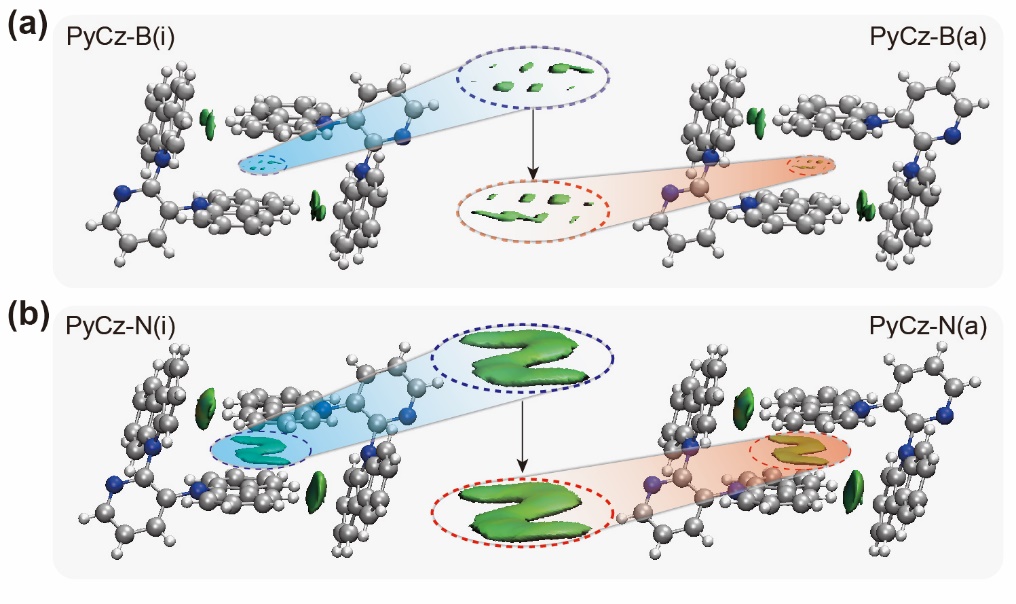
**

**Figure S16.** (a) The calculated molecular interactions (green isosurface) in dimers of PyCz-B(i) and PyCz-B(a). Note that the isovalue is 0.008. (b). The calculated molecular interactions (green isosurface) in dimers of PyCz-N(i) and PyCz-N(a). Note that the isovalue is 0.012.

**
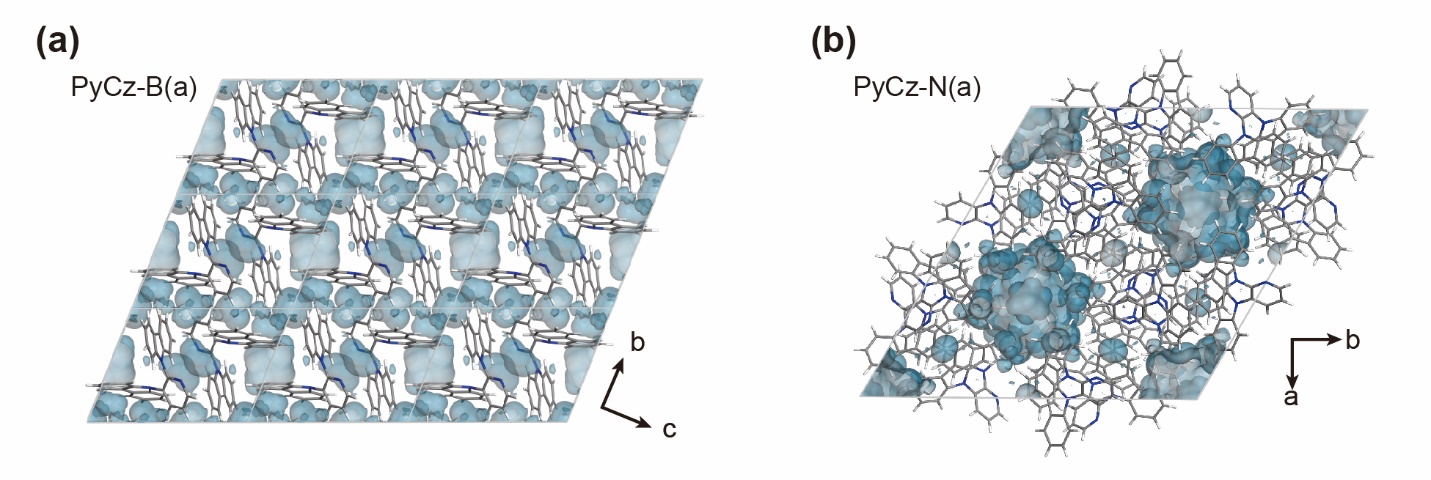
**

**Figure S17.** Free volume region in single crystal cells of PyCz-B (a) and PyCz-N (b) after long photoactivation.

**Table S11.** Calculated occupied volume, free volume as well as the proportion of free volume in PyCz-B and PyCz-N before and after long photoactivation.

| Compounds | Occupied volume (Å^3^) | Free volume (Å^3^) | Proportion (%) |
| --- | --- | --- | --- |
| PyCz-B(i) | 955.12 | 132.59 | 12.19 |
| PyCz-B(a) | 953.50 | 130.81 | 12.06 |
| PyCz-N(i) | 8441.68 | 1712.27 | 16.86 |
| PyCz-N(a) | 8350.31 | 1638.12 | 16.40 |

In order to clarify why oxygen matters more for PyCz-N than PyCz-B, the crystal packing of PyCz-N was especially studied. As shown in the Figure S18, large cavities were formed in the crystal whose maximum and minimum diameter is 9.154 Å and 3.681 Å, which is larger than the diameter of oxygen (3.46 Å). After long photoactivation, the scale of cavities is narrowed, whose maximum and minimum diameter is changed to 9.154 Å and 3.681 Å. The large cavities can generate larger contact area between the materials and oxygen. Thus, PyCz-N is more sensitive to oxygen due to the principle of oxygen quenching.

**
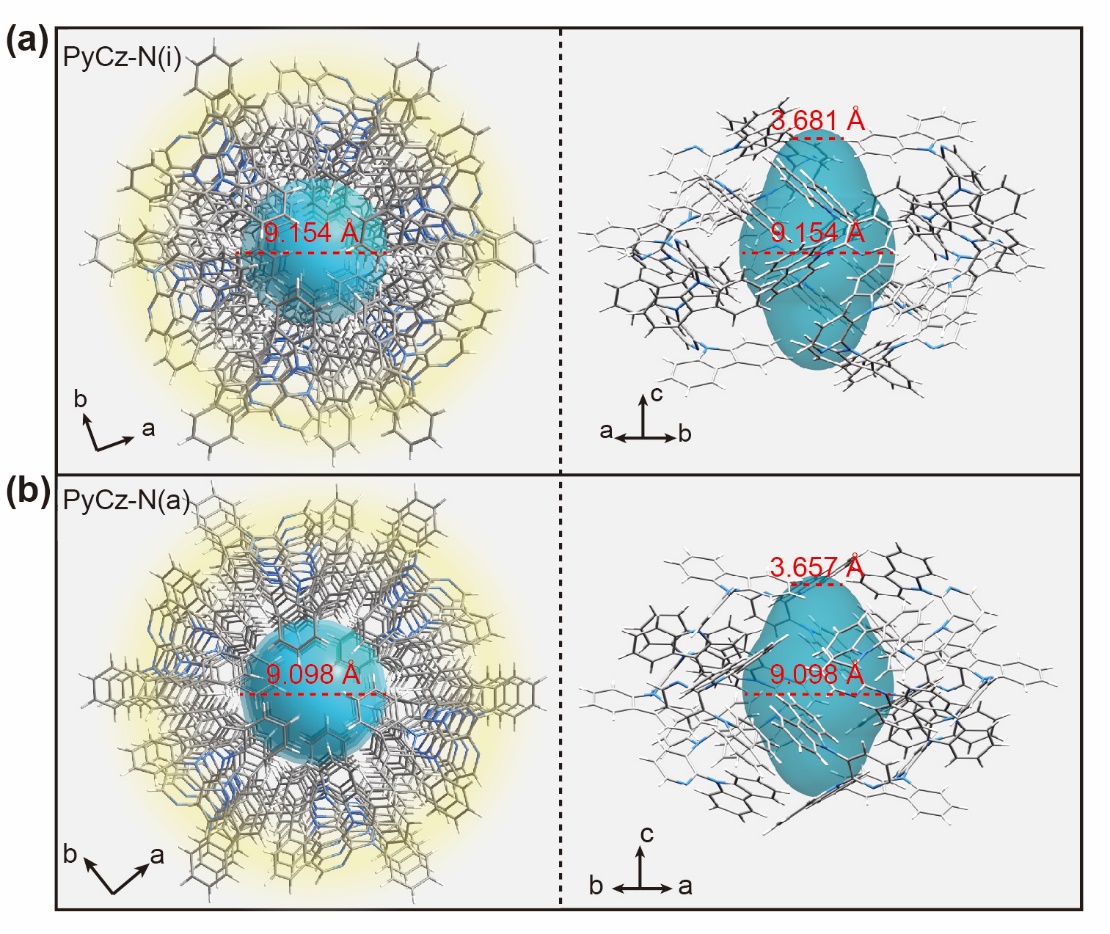
**

**Figure S18.** The cavities in PyCz-N before (a) and after long photoactivation (b).

6. Application

The photoactivated dynamic phosphorescence of PyCz-N can gradually deactivated to its initial state in ambient conditions. Thus PyCz-N can be applied to timing data storage divices, where the long photoactivation process can be regarded as data writing, instant UV light excitation can be regarded as data reading. The data can be stored before PyCz-N totally deactivating to its initial state, more than 4 h, as shown in the Figure S19. In Figure S20, a simple device was composed by 2 × 4 PyCz-N data points. In ambient conditions or under short UV light irradiation, the data can not be obtained. Through long photoactivation and gradually deactivation under ambient conditions, different information (I, A and M) can be repeatedly stored in the device with corresponding ACS II codes (01001001, 01000001 and 01001101). The information can be read within 6 h after long photoactivation and the device can be rewritten by other information after complete deactivation.

**
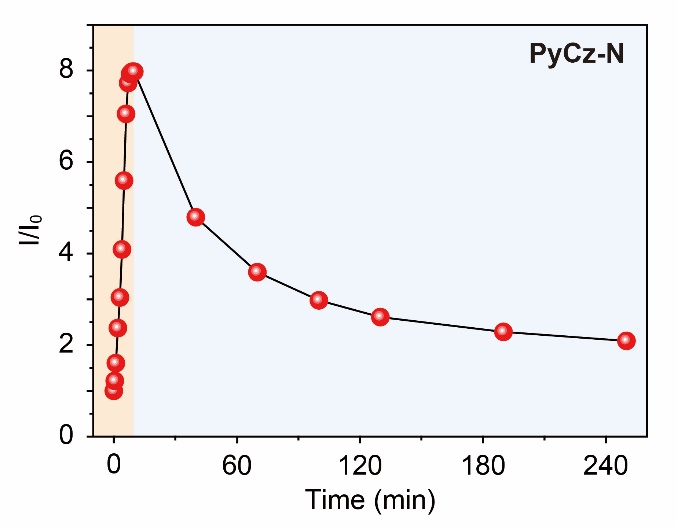
**

**Figure S19.** The relative intensity (I/I_0_) of 544 nm UOP of PyCz-N as a function of long photoactivation and deactivation time.

**
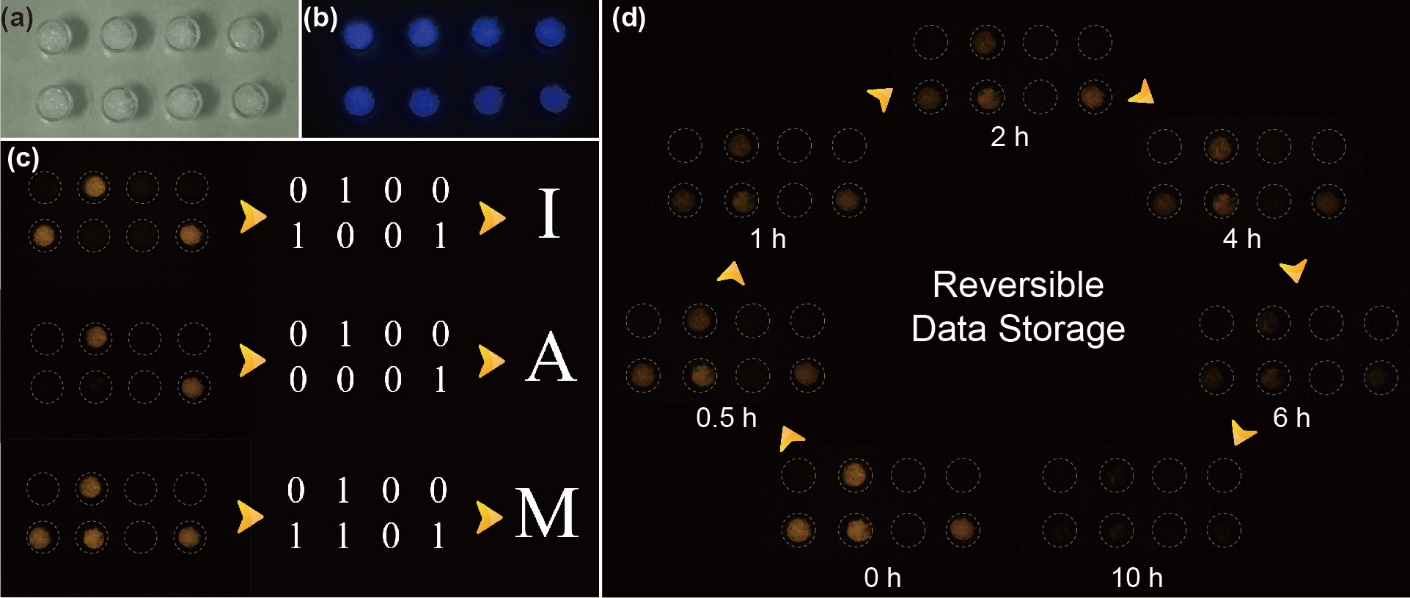
**

**Figure S20.** The application of PyCz for rewritable timing data storage. The data storage device under room light (a) and 365 nm UV light irradiation (b). (c) Demonstration of visual data storage based on PyCz-N. Note that the visual state of UOP is defined as 1. The dark state of UOP is defined as 0. (d). The reversibility of the rewritable visual data storage.

7. Supporting movies

The supplementary videos were recorded by Nikon EOS 700D in dark under ambient conditions.

**Movie S1.** This video demonstrates the process of long photoactivation of PyCz-B and PyCz-N under the irradiation of a strong UV lamp with the power of 40 mW/cm^2^. PyCz-B was easily activated within 1~2 s. For PyCz-N, it needed more than 8 min to be activated.

**Movie S2**. This video demonstrates the afterglow of PyCz-N under different temperature (273, 253, 233, 213, 193 and 77 K). In 273 K, the after emission of PyCz-N can not be observed by naked eyes. With the temperature decreased, the PyCz-N showed gradual increase in lifetime.

**Movie S3 and Movie S4.** These videos demonstrate the rewritable visual data storage based on PyCz-N phosphor. As shown in Movie S3, with strong UV light irradiation, different information (I, A and M) can be stored with corresponding ACS II codes (01001001, 01000001 and 01001101). In Movie S4, it is found that the information of “M” can be stored for about 6 hours owing to the deactivation behavior of the phosphor. Then the device can also be rewritten with other information with UV light.

8. References

1 C. Tang, R. Bi, Y. Tao et al., “A versatile efficient one-step approach for carbazole–pyridine hybrid molecules: highly efficient host materials for blue phosphorescent OLEDs,” *Chemical Communication*, vol. 51, no. 9, pp. 1650-1653, 2015.

2 L. Gu, H. Shi, M. Gu et al., “Dynamic Ultralong Organic Phosphorescence by Photoactivation,” *Angewandte Chemie International Edition*, vol. 57, no. 28, pp. 8425-8431, 2018.
